# Chronic exposure to complex metal oxide nanoparticles elicits rapid resistance in *Shewanella oneidensis* MR-1†
†Electronic supplementary information (ESI) available: Additional data figures and methods. See DOI: 10.1039/c9sc01942a


**DOI:** 10.1039/c9sc01942a

**Published:** 2019-08-30

**Authors:** Stephanie L. Mitchell, Natalie V. Hudson-Smith, Meghan S. Cahill, Benjamin N. Reynolds, Seth D. Frand, Curtis M. Green, Chenyu Wang, Mimi N. Hang, Rodrigo Tapia Hernandez, Robert J. Hamers, Z. Vivian Feng, Christy L. Haynes, Erin E. Carlson

**Affiliations:** a Department of Chemistry , University of Minnesota , 207 Pleasant St. SE , Minneapolis , MN 55455 , USA . Email: carlsone@umn.edu; b Department of Biochemistry, Molecular Biology, and Biophysics , University of Minnesota , 321 Church Street SE , Minneapolis , Minnesota 55454 , USA; c Chemistry Department , Augsburg University , 2211 Riverside Ave , Minneapolis , MN 55454 , USA; d Department of Chemistry , University of Wisconsin-Madison , 1101 University Avenue , Madison , WI 53706 , USA; e Department of Medicinal Chemistry , University of Minnesota , 208 Harvard Street SE , Minneapolis , 55454 , USA

## Abstract

Engineered nanoparticles are incorporated into numerous emerging technologies because of their unique physical and chemical properties. Many of these properties facilitate novel interactions, including both intentional and accidental effects on biological systems. Silver-containing particles are widely used as antimicrobial agents and recent evidence indicates that bacteria rapidly become resistant to these nanoparticles. Much less studied is the chronic exposure of bacteria to particles that were not designed to interact with microorganisms. For example, previous work has demonstrated that the lithium intercalated battery cathode nanosheet, nickel manganese cobalt oxide (NMC), is cytotoxic and causes a significant delay in growth of *Shewanella oneidensis* MR-1 upon acute exposure. Here, we report that *S. oneidensis* MR-1 rapidly adapts to chronic NMC exposure and is subsequently able to survive in much higher concentrations of these particles, providing the first evidence of permanent bacterial resistance following exposure to nanoparticles that were not intended as antibacterial agents. We also found that when NMC-adapted bacteria were subjected to only the metal ions released from this material, their specific growth rates were higher than when exposed to the nanoparticle. As such, we provide here the first demonstration of bacterial resistance to complex metal oxide nanoparticles with an adaptation mechanism that cannot be fully explained by multi-metal adaptation. Importantly, this adaptation persists even after the organism has been grown in pristine media for multiple generations, indicating that *S. oneidensis* MR-1 has developed permanent resistance to NMC.

## Abstract 2

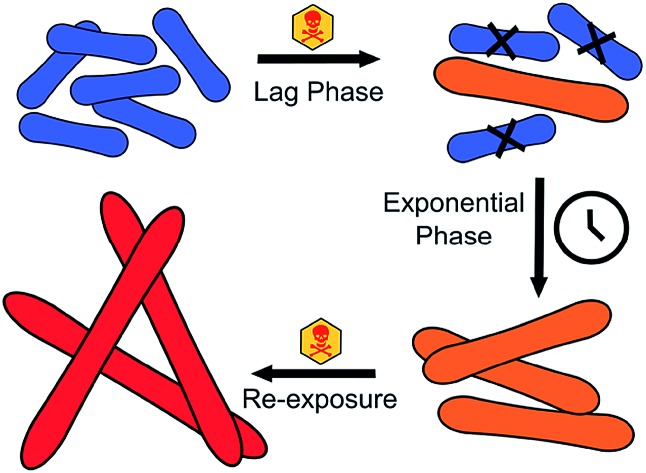
We provide the first evidence of permanent bacterial resistance following exposure to nanoparticles that were not intended as antibacterial agents.

## 

Engineered nanomaterials have been impactful in a wide range of emerging technologies and materials (*e.g.* textiles,^1^,^2^ electronic screens,^3^ and environmental remediation^4^) due to their unique physical and chemical properties. These characteristics also contribute to their antimicrobial properties. Metal and metal oxide nanoparticles such as silver,^5^,^6^ zinc oxide,^7^ and copper oxide^8^ have been used as antibacterial agents;^9^ the toxicity of these materials is strongly correlated to their size,^10^,^11^ dissolution,^10^,^12^,^13^ and composition,^14^ and the native susceptibility of the organism. Even nanomaterials not intended for antimicrobial applications (zerovalent iron for environmental remediation,^15^ carbon nanotubes,^16^ and CdSe/ZnS quantum dots^17^) can be toxic to bacteria. This toxicity occurs through mechanisms such as reactive oxygen species (ROS) generation, metal dissolution, lipid peroxidation, and mechanical stress.^18^ Another such nanoparticle is lithiated nickel manganese cobalt oxide (Li_*x*_Ni_*y*_Mn_*z*_Co_1–*y*–*z*_O_2_, NMC), a battery cathode material that is used at large-scale in electric vehicles.^19^ In January 2015, there were more than 740 000 electric vehicles in operation and it is estimated that by 2020 there will be several million electric vehicles on the road, each containing ∼50 kg of nanoscale cathode materials.^14^,^20^ Nanoparticles have a large surface-area-to-volume ratio, which is advantageous in materials such as NMC, where lithium is able to efficiently shuttle in and out of the cathode, resulting in increased rate capability. However, some of the properties of NMC that make it an attractive battery cathode are also responsible for its toxicity to bacteria, such as *Shewanella oneidensis* MR-1.^12^,^14^

*S. oneidensis* MR-1 is a ubiquitous, Gram-negative, soil-, sediment-, and aquatic-dwelling bacterium that plays a critical role in environmental metal-cycling and is therefore a commonly used model organism in environmental studies.^21^–^23^ The redox capabilities of *S. oneidensis* MR-1, especially related to the element manganese, indicate that it would be present in environments that may be contaminated by nanoparticle pollutants, such as NMC.^24^*Shewanella* has been found to have important roles in metal-cycling in freshwater systems, despite low abundance, and is found in fish making it the prominent cause of fish spoilage.^25^,^26^ Previous work has shown that modest concentrations of NMC (less than 25 mg L^–1^) impaired the growth of *S. oneidensis* MR-1 to the point that respiration and turbidity were undetectable.^12^ Upon exposure to NMC (5 mg L^–1^), bacterial cultures had extended lag periods (∼20 h), but could ultimately recover to the same level of total respiration as an unexposed population. This led us to develop two hypotheses about the process of bacterial recovery: either (1) the toxicity of NMC is limited both temporally and spatially (*i.e.* aggregation and sedimentation after a period of time or the accumulation of a biomolecular corona on the nanoparticles), so that the nanoparticles become unavailable to the bacteria and an unaffected population would grow stochastically. The growth delay is due to a smaller starting population, or (2) the delay is due to the time required for a subpopulation of bacteria to adapt to NMC toxicity and recover. Given the serious implications of permanently altering bacterial behaviors, as is most widely understood in relation to antibiotic resistance, we sought to investigate the nature of this phenomenon further.

Toxicity studies are most commonly performed with short-term, high-dose exposures. These studies enable rapid determination of the acute effects of a substance, but do not reveal the complete extent of their impact. Chronic exposures are critical in ecological toxicity investigations since pollutants such as metals and antibiotics remain in the environment for extended periods. Long-term exposure experiments can reveal deeper complexities of the toxicity and multi-generational impacts.^27^ This is especially true in the study of bacterial exposures, as these organisms replicate very quickly and can share genetic information, which enables them to rapidly adapt to changes in their local environment. It is well-known that repeated exposure to antibiotics, even below the minimum inhibitory concentration (MIC), can stimulate resistance in bacteria or change the diversity of bacterial communities.^28^,^29^ Therefore, it is important to consider both the environmental relevance and the adaptation capabilities of bacteria when designing toxicity experiments and to perform chronic exposures to evaluate the full extent of organismal response.

In this study, we expose *S. oneidensis* MR-1 to NMC for multiple generations to simulate a chronic, environmental exposure. This resulted in the rapid adaptation of the bacteria to both the nanoparticle and the metal ion controls used to mimic nanoparticle dissolution. We also found that there is a nanoparticle-specific impact based on the growth and morphology of the bacteria that cannot be accounted for by metal dissolution and that would not have been discovered without chronic exposure experiments. Thus, it is clear that even nanoparticles that have been developed exclusively for technological applications, such as NMC, may dramatically affect environmental organisms should they be released accidentally or through improper disposal.

## Results & discussion

### Impact of initial NMC exposure on *Shewanella oneidensis* MR-1 (Passage A)

**Fig. 1:**
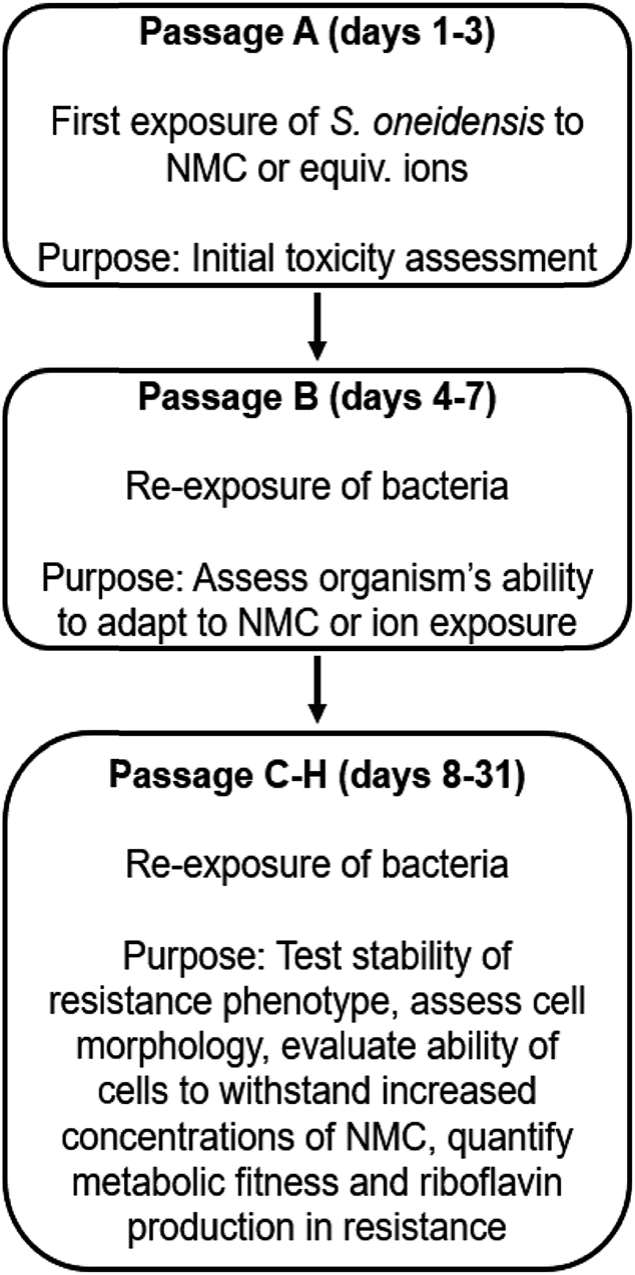
Schematic of experiments performed in this work to assess *S. oneidensis* MR-1 resistance to NMC. A more detailed schematic is provided in Fig. S3.†

**Fig. 2:**
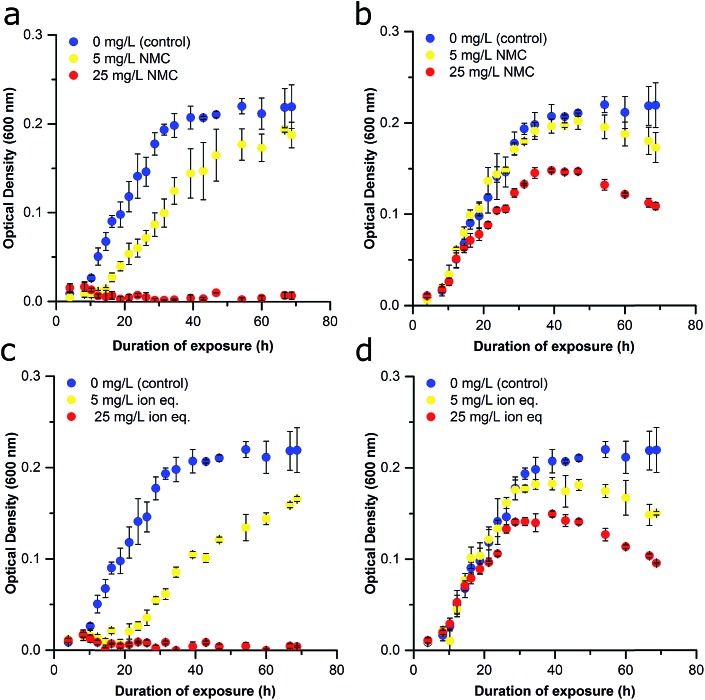
Effect of delayed NMC and ion exposure on growth inhibition of *S. oneidensis* MR-1 (Passage A). (a) Bacterial exposure at the time of inoculation to no NMC (blue), 5 mg L^–1^ NMC (yellow), and 25 mg L^–1^ NMC (red). (b) Bacterial exposure 10 h after the inoculation to no NMC (blue), 5 mg L^–1^ NMC (yellow), and 25 mg L^–1^ NMC (red). (c) Bacterial exposure to constitutive ions of NMC at time of inoculation: no NMC ions (blue), ions of 5 mg L^–1^ eq. of NMC (yellow), and ions of 25 mg L^–1^ eq. of NMC (red). (d) Bacterial exposure to constitutive ions of NMC 10 h after inoculation: no NMC ions (blue), ions of 5 mg L^–1^ eq. of NMC (yellow), and ions of 25 mg L^–1^ eq. of NMC (red). Error bars represent the standard deviation of three replicates.

NMC was synthesized with a specific stoichiometry of 1 : 1 : 1 Ni : Mn : Co, which is the most toxic NMC studied to date.^14^ TEM images reveal the hexagonal, sheet structures of the NMC nanoparticles with a size distribution across the planar surface of 84 ± 22 nm (Fig. S1†). The impact of NMC on *S. oneidensis* MR-1 was evaluated by optical density (OD) at 600 nm as a measure of population density. Nanoparticle exposures were performed in minimal media to more closely represent an environmental water system while limiting external influences that may also affect nanoparticle–nanoparticle or nanoparticle–bacteria interactions as compared to nutrient rich medias such as LB. NMC suspended in minimal media exhibit a *ζ*-potential of –10.3 ± 0.547 mV indicating that the nanoparticles are not colloidally stable. Dynamic light scattering (DLS) further revealed that NMC begins to aggregate substantially after 24 h (Fig. S2†). Initially, bacteria were exposed to NMC immediately upon inoculation of the culture (time 0 h), yielding growth curves similar to those previously published (Fig. 2A; brief experimental scheme provided in Fig. 1, detailed experimental scheme provided in Fig. S3†).^12^ Cultures were exposed to either 5 mg L^–1^ or 25 mg L^–1^ NMC, concentrations that previous research indicate are representative of recoverable nanoparticle pressure (5 mg L^–1^) and irrecoverable nanoparticle toxicity (25 mg L^–1^).^12^,^14^ Immediate exposure to 25 mg L^–1^ NMC rapidly killed the organisms, making it difficult to assess the potential long-term effects of chronic exposure. Instead, we found that allowing the culture to grow before addition of NMC (10 h) enabled us to perform chronic exposures and to observe more subtle effects than cell death. The delay in NMC addition could also facilitate examination of a greater range of NMC concentrations since the cultures are less sensitive to 25 mg L^–1^ NMC (Fig. 2B). This change in response is likely due to the inoculum effect, a phenomenon often used to describe the impact of bacterial density on MIC values, where a higher bacterial density requires a higher concentration of antibiotic to kill the bacteria. Although traditionally used to discuss changes in antibiotic toxicity, it could also explain the observed changes in nanoparticle toxicitiy.^30^ More specifically, this effect could indicate that NMC has different mechanisms of toxicity at different growth stages.^31^ Interestingly, previous work demonstrated that immediate exposure caused an increase in the lag phase; however, we found that delayed exposure (10 h, 25 mg L^–1^ NMC) affected the maximal population density achieved in the stationary phase by at least half in comparison to untreated cultures. Studies utilizing similar delayed exposure protocols have also yielded a decrease in the stationary phase when *S. oneidensis* MR-1 was exposed to chromium(vi) and *E. coli* to silver nanoparticles (AgNPs).^31^,^32^

Previous work has shown that NMC toxicity to *S. oneidensis* MR-1 is related to its dissolution and release of ions, particularity nickel and cobalt, into the growth media.^12^ Although some of these metals are micronutrients, metal homeostasis of the organism is disturbed at higher concentrations of nickel and copper, which could cause oxidative stress, the replacement of the native metal cofactor of some proteins, or the binding of metals with critical functional groups on proteins or nucleic acids.^18^,^33^–^35^ Delayed exposures were also performed with solutions of LiOH, NiCl_2_, MnSO_4_, CoCl_2_ to recapitulate the known metal ion concentrations after 96 h of NMC dissolution based on ICP-OES measurements (Fig. S4†). These too revealed the dose-dependent nature of NMC-derived ion toxicity and strengthened the postulation that dissolved ions are responsible for a large proportion of NMC toxicity to *S. oneidensis* MR-1 (Fig. 2C and D).^12^,^14^ Comparison of the growth curves of bacteria exposed to NMC or the ion equivalent in Passage A reveal only subtle differences (*e.g.*, lag time (*λ*), specific growth rate (*μ*), maximum OD; Fig. 2B, D and S5†).

### Repetitive exposure to NMC (Passage B)

**Fig. 3:**
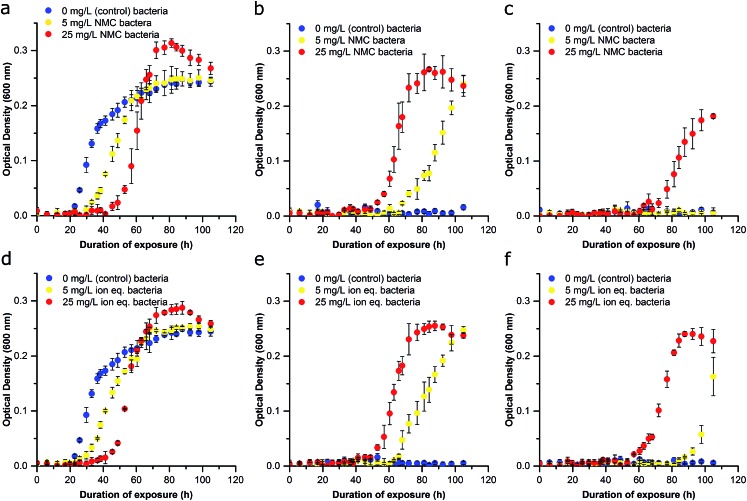
Effect of repetitive NMC and ion exposure on growth inhibition of *S. oneidensis* MR-1 (Passage B). (a–c) Bacteria from Passage A that were previously exposed to control conditions in Passage A are represented in blue, bacteria that were exposed to 5 mg L^–1^ NMC in Passage A are represented in yellow, and bacteria exposed to 25 mg L^–1^ NMC in Passage A are represented in red. (a) Bacteria from Passage A cultured in control conditions, (b) bacteria from Passage A cultured in 5 mg L^–1^ NMC, (c) bacteria from Passage A cultured in 25 mg L^–1^ NMC. (d–f) Bacteria from Passage A that were previously exposed to control conditions in Passage A are represented in blue, bacteria that were exposed to 5 mg L^–1^ NMC ion eq. in Passage A are represented in yellow, and bacteria exposed to 25 mg L^–1^ NMC ion eq. in Passage A are represented in red. (d) Bacteria from Passage A cultured in control conditions, (e) bacteria from Passage A cultured in 5 mg L^–1^ NMC ion eq., (f) bacteria from Passage A cultured in 25 mg L^–1^ NMC ion eq. Error bars represent the standard deviation of three replicates.

To examine the ability of this organism to activate processes required for survival under toxic conditions, we performed serial nanoparticle exposures and monitored bacterial response (Fig. 1). After an initial 72 h of growth (preventing cultures from reaching declining stage), cultures exposed to 0, 5, or 25 mg L^–1^ NMC (Passage A) were each diluted to the same OD (∼0.1; half of the starting bacterial density used in Passage A) to achieve similar population densities and further diluted 1 : 10 (v/v) into fresh media and lactate for Passage B. Freshly inoculated cultures generated from Passage A bacteria were immediately exposed (0 h) to either 5 mg L^–1^, 25 mg L^–1^ NMC or left untreated for 96 h. Unperturbed *S. oneidensis* had a doubling time of ∼6 hours in minimal media. Thus, there are ∼16 generations of replication per 96 h passage. Cultures reseeded into fresh media without nanoparticles (0 mg L^–1^) were able to reach exponential phase growth in the order of apparent fitness; the control cultures had the shortest lag time, followed by the cultures previously exposed to 5 mg L^–1^ NMC, and then those previously exposed to 25 mg L^–1^ NMC (lag time doubled in comparison to control). The specific growth rate for cultures exposed to 25 mg L^–1^ NMC in Passage A was less than half that of the control samples (Fig. 3A and S5†). When Passage A cultures were reseeded and exposed to 5 mg L^–1^ NMC, only the cultures that had previously been exposed to 5 mg L^–1^ or 25 mg L^–1^ NMC were able to grow (Fig. 3B). These results indicate that due to the initial exposure, *S. oneidensis* MR-1 has adapted and is able to replicate under conditions that were previously toxic.

Additionally, organisms that previously experienced the highest concentrations of NMC appeared to be the most robust in subsequent exposures. For example, when the Passage A cultures were cultivated in media containing 25 mg L^–1^ NMC for Passage B, only bacteria that had previously been subjected to 25 mg L^–1^ NMC could survive (Fig. 3C). This adaptation is significant as the bacteria are capable of growth in NMC concentrations that were lethal to unexposed bacteria (Fig. 2A). Adaptation was rapid and occurred in around 6 generations (with an estimated doubling time of 11.5 h of bacteria exposed to 25 mg L^–1^ NMC during Passage A exposure). This is considered rapid compared to other work, which showed that *E. coli* became resistant to AgNP exposure in 100 generations, *B. subtilis* adapted to a concentration of nanosilver(i) oxide that was 1.5× greater than the lethal dose in 13 days, while others indicated an increase in MIC after only a few sub-culturing periods of 24 h.^36^–^38^ These experiments were also performed by reseeding cultures that had been exposed to ions in Passage A into fresh, ion-containing media, with concentrations representing the dissolution of 5 mg L^–1^ and 25 mg L^–1^ NMC after 96 h (Fig. 3D–F). The trend in these growth curves is similar to that of the NMC exposures but indicates that the ions may be less toxic than the nanoparticle when comparing adapted cultures (compare Fig. 3C, F and S5†).^14^

### Adaptation characterization (Passage C, D, and beyond)

**Fig. 4:**
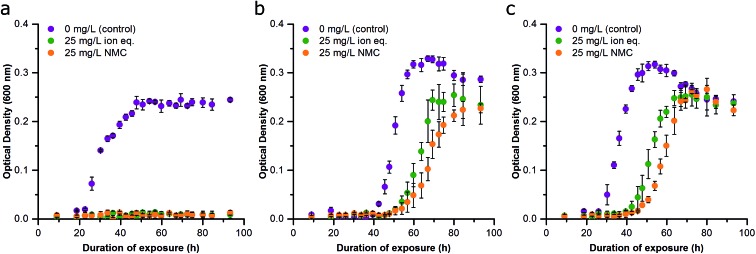
Effect of repetitive NMC and ion exposure on growth inhibition of *S. oneidensis* MR-1 (Passage C). (a) Bacteria cultured for two passages in pristine media exposed to no NMC (purple), 25 mg L^–1^ NMC ion eq. (green), and 25 mg L^–1^ NMC (orange). (b) Bacteria cultured for two passages with 25 mg L^–1^ NMC, which was then exposed to no NMC (purple), 25 mg L^–1^ NMC ion eq. (green), and 25 mg L^–1^ NMC (orange). (c) Bacteria cultured for two passages with 25 mg L^–1^ NMC ion eq., which was then exposed to no NMC (purple), 25 mg L^–1^ NMC ion eq. (green), and 25 mg L^–1^ NMC (orange). Error bars represent the standard deviation of three replicates.

The adaptation phenotype was characterized by growth studies in addition to analysis of population respiration. For Passage C, the adaptation to NMC toxicity and constitutive ion toxicity were compared. The untreated populations and those that had been exposed to 25 mg L^–1^ NMC and the ion equivalent of 25 mg L^–1^ for two passages were diluted and re-exposed to 25 mg L^–1^ NMC, the 25 mg L^–1^ ion equivalent, or control media. The concentration of 25 mg L^–1^ was selected because it represents a lethal dose of NMC and its ion equivalent to sensitive *S. oneidensis* MR-1. Optical density studies reveal that when untreated cultures were reseeded into fresh, NMC-, and ion-containing media, the population of *S. oneidensis* MR-1 was only able to replicate in clean media as expected (Fig. 4A). Again, we found that ion- and NMC-adapted cultures had shorter lag periods in control conditions than when the same organisms were grown in the presence of NMC or ions (Fig. 4B, C and S5†). Interestingly, when the population adapted to 25 mg L^–1^ NMC was exposed to the equivalent metal ions, the specific growth rate was higher than the culture that was instead exposed to NMC (Fig. 4B and S5†). This was also seen when the population adapted to metal ions (equivalent to 25 mg L^–1^ NMC) was exposed to ions or NMC (Fig. 4C). The dissolution of metal ions has been proposed to be the major mechanism of NMC antibacterial activity to *S. oneidensis* MR-1, and comparison of NMC- and ion-adapted cultures confirm this. However, in both treatments of adapted cultures, exposure to NMC presented a greater challenge to bacterial growth than the constitutive ions based on specific growth rate differences, indicating that adaptation to nanoparticles is more complex than adaptation to multiple metal ions alone (Fig. S5†).^14^,^39^,^40^

The distinction between the toxicity of the nanoparticle or its ions was difficult to discern during the first exposure in Passage A, but comparison of the adapted cultures to unadapted organisms made these differences more apparent. We had anticipated that exposure to the entirety of NMC dissolution products (the metal ion control) immediately after culture inoculation would be more toxic than NMC due to the heavy front-loading of the free ions on an unestablished, low-density culture. Yet, there is more available lactate for metal chelation, based on previous modeling work, which in turn could make the toxic metal ions less bioavailable.^12^,^14^,^34^ As these two effects cannot be easily reconciled, this is most likely an indication of unique particle-specific toxicity, such as ROS generation.^14^,^41^

**Fig. 5:**
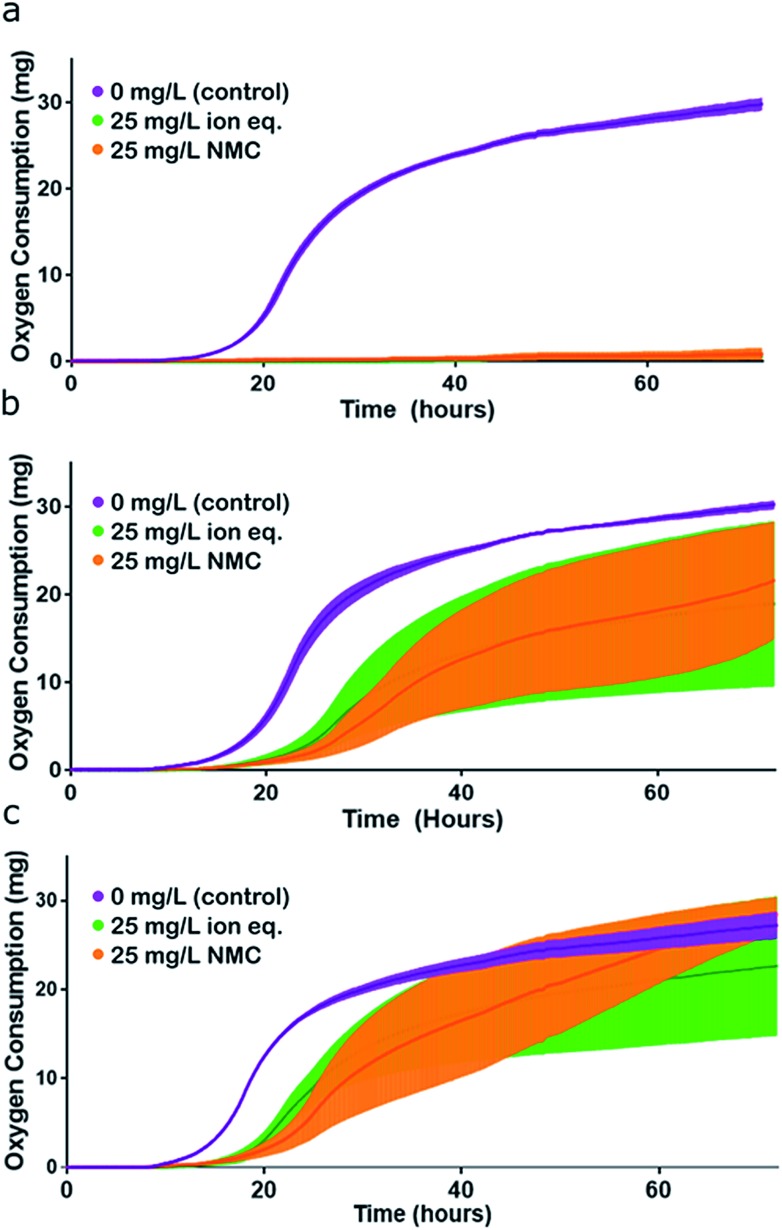
Assessment of organismal fitness by measurement of oxygen consumption. (a) Respirometry curves of *S. oneidensis* MR-1 control (unadapted) cultures exposed to control conditions (purple), 25 mg L^–1^ NMC (orange), and 25 mg L^–1^ NMC ion eq. (green) in Passage E. (b) Respirometry curves of NMC-adapted cultures exposed to control conditions (purple), 25 mg L^–1^ NMC (orange), and 25 mg L^–1^ NMC ion eq. (green) in Passage E. (c) Respirometry curves of ion-adapted cultures exposed to control conditions (purple), 25 mg L^–1^ NMC (orange), and 25 mg L^–1^ NMC ion eq. (green) in Passage E. Error bars represent the standard deviation of replicates. Representation of this figure without standard deviations is located in Fig. S7.†

We tested the consistency of this trend by performing the same experiment in Passage E with cultures that had been continuously cultured with either 25 mg L^–1^ NMC, 25 mg L^–1^ ion equivalent, or in control media for the previous four passages. The same trend was observed in optical density growth curves (Fig. S6†). In this passage, turbidity measurements were supplemented with respirometry measurements (cumulative O_2_ consumption) of the cultures, which also demonstrated the same trend in toxicity and adaptation (Fig. 5). Analysis of the first derivative of these curves reveals differences in the time required to reach peak oxygen consumption (Fig. S7†). The organisms that were unexposed in Passage E reached peak oxygen consumption first, followed by the bacteria that had been exposed to ions, and finally, cultures that had previously been subjected to NMC took the longest to reach their peak respiration rate. These data also indicate that exposed organisms are respiring less overall than control bacteria in pristine media.

**Fig. 6:**
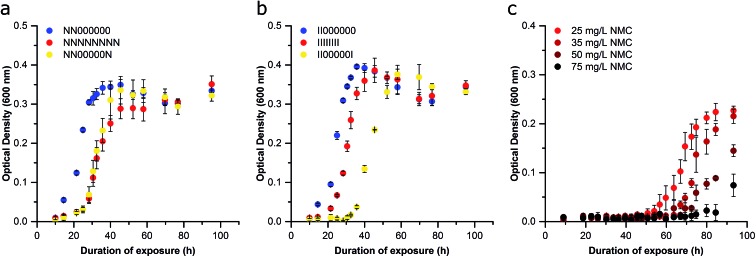
Examination of the stability of the bacterial adaptation following a period of non-exposure (Passage H). (a) Bacteria cultured for two passages in 25 mg L^–1^ NMC were then grown for 5 passages without exposure, and then a final passage exposed to 25 mg L^–1^ NMC (NN00000N; yellow) or no NMC (NN000000; blue) and compared to continually exposed cultures (NNNNNNNN; red). (b) Bacteria cultured for two passages in the ion eq. of 25 mg L^–1^ NMC were then for grown for 5 passages without exposure, and then a final passage exposed to the ion eq. of 25 mg L^–1^ NMC (II00000I; yellow) or no NMC (II000000; blue) and compared to continually exposed cultures (IIIIIIII; red). (c) Bacteria cultured for two passages in 25 mg L^–1^ NMC were then exposed to increasing concentrations of NMC (Passage C). Error bars represent the standard deviation of three replicates.

We next sought to determine if the organismal adaptation(s) were stable, which would imply a genome-level alteration was facilitating resistance. First, cells were removed from NMC or ion pressure and cultured in fresh media with no treatment starting with Passage C for subsequent passages in an attempt to rescue the sensitive phenotype. After each passage was grown under normal conditions (unexposed), the adapted cultures were again exposed to 25 mg L^–1^ NMC or the ion equivalent. These cultures were compared to the control, NMC-, and ion-adapted cultures that had been repeatedly exposed throughout all passages. After five additional passages (estimated to be 67 generations) with no treatment, the NMC- and ion-adapted cultures maintained their adaptation phenotype, indicating that this adaption is stable in the absence of NMC or metal ion pressure (Fig. 6A and B). This likely indicates a chromosomal mutation that does not perturb organism fitness, which will be assessed in future studies.^42^ This is also implied by the exposed organism's ability to achieve similar specific growth rates and lag times in comparison to control (Passage D and beyond; Fig. S5 and S8†). Permanent perturbations to bacterial characteristics have the potential to change the behavior of a microbial community and therefore have significant and long-term impact on ecosystems health and stability. Microbes perform a wide range of functions in the environment such as nutrient cycling, which make them excellent indicators of environmental health.^43^,^44^

To demonstrate that the adapted organism could survive under increasing pressure, bacteria that had been exposed to 25 mg L^–1^ NMC (Passage B) were reseeded into higher concentrations of NMC, revealing that the organism could now grow in 75 mg L^–1^ NMC (3× the previous exposure; Fig. 6C). We observed an increase in the lag phase at higher NMC concentrations and hypothesize that this either represents the activation of additional molecular defenses (*e.g.* efflux pumps, oxidative stress protection), increased preliminary killing that decreases the initial cell density, or the development of new mutations. An increase in lag phase has been associated with adaptation to new environments, as well as to toxic substances such as antibiotics. Although little is known about bacterial lag phase, it is likely that an increased lag phase enables increased tolerance to antibiotics to promote the further evolution of antibiotic resistance.^45^ Other work has also shown an increase in lag phase when bacteria where exposed to metal oxide nanoparticles.^46^,^47^ In Passage D, the population that grew in 50 mg L^–1^ NMC was successfully cultured in 100 mg L^–1^ NMC (Fig. S9†). This confirms that the adaption is robust and flexible as the *S. oneidensis* MR-1 is capable of replication in a concentration of NMC that is 20 times that which was found to kill unexposed bacteria.

To quantify the increased tolerance of the organism to NMC, we obtained the MIC values for lithium, nickel, manganese, and cobalt ions on both the adapted and control populations in Passage D, as the ability to withstand increased concentrations of an antibacterial substance is one of the hallmarks of resistance.^38^,^48^ The adapted bacteria were capable of surviving in concentrations of nickel and cobalt metal ions at least three times higher than the unadapted cultures (Fig. S10†). No changes in the MIC values for dissolved lithium and manganese were observed, likely because the concentrations required to cause adaptation pressure are not achieved through NMC dissolution.

The final confirmation of resistance is to eliminate the possibility that there was a subpopulation of persistent microbes in the original culture. Because we see active replication of the adapted populations and those exposed to increasing concentrations of NMC, it is likely that the bacteria are not persistent. As further confirmation, we performed a minimum duration for killing 99% (MDK99) assay on the unadapted *S. oneidensis* MR-1, which provides an indication of the mechanism of bacterial adaptation [colony forming units (CFUs) per mL]. This assay revealed that there is no persistent or resistant subpopulation in the unadapted population (Fig. S11†). Additionally, unadapted *S. oneidensis* exposed to 25 mg L^–1^ NMC, which appears to cause complete cell death, were reseeded into nutrient-rich lysogeny broth (LB). There was no growth in this subculture, which indicated that there are no viable bacteria after exposure to this dose of NMC (data not shown). In combination, these three observations, adaptation stability, increased MIC values, and the lack of persistent population, indicate that *S. oneidensis* MR-1 has developed stable resistance to NMC, permanently altering its biochemical and morphological characteristics (*vide infra*).^38^,^49^

### Initial mechanistic investigation of resistance: electron microscopy

**Fig. 7:**
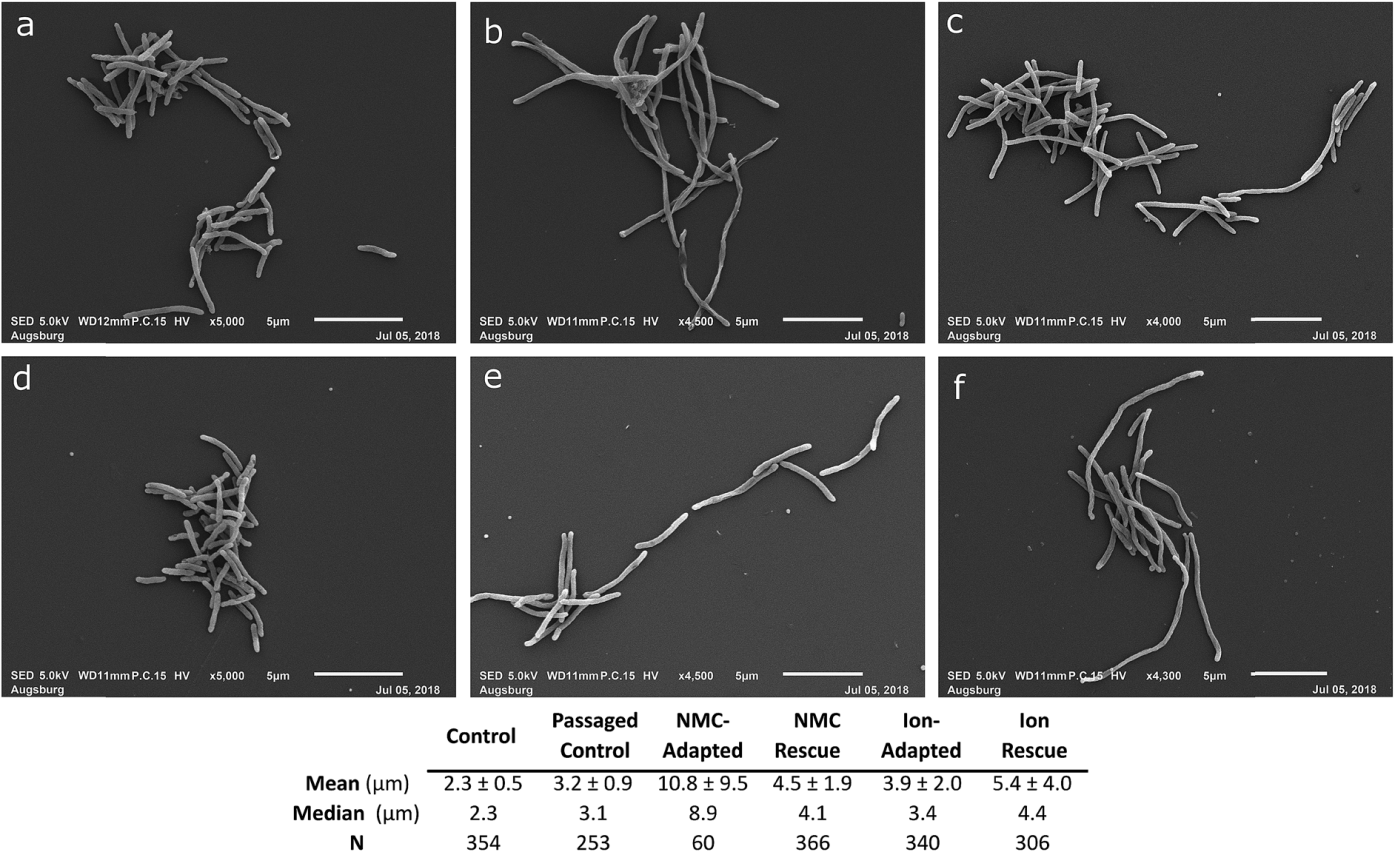
Morphology assessment. Scanning electron micrographs of *S. oneidensis* MR-1 from Passage D exposed to (a) no nanoparticle or ions over 4 passages, (b) 25 mg L^–1^ NMC over 4 passages (c) 25 mg L^–1^ NMC ion eq. over 4 passages (d) no nanoparticle or ions for one passage, (e) 25 mg L^–1^ NMC over 2 passages followed by 2 passages without exposure, (f) 25 mg L^–1^ NMC ion eq. over 2 passages followed by 2 passages without exposure. Table indicates mean, median, and standard deviation in μm for the imaged microbe populations. Statistical analyses to compare the lengths of the bacteria are included in the ESI (Fig. S12).†

We next examined the morphology of cultures that were resistant to the ions and NMC nanoparticles in comparison to the passaged control with scanning electron microscopy (SEM; Passage D; Fig. 7 and S12†). The bacteria that had adapted to NMC exposure were filamented compared to the passaged control, showing a massive range of lengths up to 60 μm (median length 8.9 μm, passaged control 3.1 μm). The NMC-adapted bacteria (median 8.9 μm) were also substantially longer than ion-adapted bacteria (median 3.4 μm), indicating there was additional burden on the nanoparticle-exposed population. The control bacteria that had been cultured for four consecutive passages were compared to a fresh culture that had never been subcultured, confirming minimal filamentation stress due to subculturing alone (median 2.3 *versus* 3.1 μm).

Filamentation is a known mechanism of bacterial response and adaptive processes as it occurs when bacteria experience stress or environmental change, and is related to some resistance mechanisms.^50^,^51^ This could give bacteria an evolutionary advantage in combatting stress and preventing further damage. The filamentation of *S. oneidensis* MR-1 under exposure conditions indicates that this is a part of its adaptation mechanism to protect from NMC toxicity and has been previously observed after chromium(iv) and cadmium selenide quantum dot exposure in *S. oneidensis* MR-1.^52^,^53^ Interestingly, when the ion-adapted population is grown in pristine media, it still shows a small increase in filamentation compared to the control, indicating that this phenotype persists even after the stress has been removed. When the NMC-adapted population is grown under control conditions, it shows a significant decrease in filamentation (now similar to ion-adapted), indicating a decrease in stress while the adaptation-phenotype persists in OD studies (Fig. 6A and 7).

Extensive bacterial filamentation may have profound impacts on the environment due to the critical role and pervasive nature of *S. oneidensis* MR-1. This stress response could influence the activity of *S. oneidensis* MR-1 in the environment due to changes in metabolic activity and metal and small molecule turnover, but could also make it more difficult for predators to consume. Finally, if filamentation is a global response to nanoparticle adaption, this could also enable pathogens to become more resistant to antibiotics as filamentation is a known antibiotic resistance mechanism.^50^,^51^

Bacteria from Passage C were also analyzed by transmission electron microscopy (TEM) to determine if cell envelope structures were altered during the adaptation process to either ions or particles (Fig. S13†). The envelope of bacteria and its components (*e.g.*, lipopolysaccharides) are the first interface of the organism to interact with the nanoparticle and its ions.^54^,^55^ Previous work has shown that there is no internalization of NMC by *S. oneidensis* MR-1 and no significant membrane association of NMC after 30 min of exposure.^12^,^14^ The negative charge of the nanoparticles makes it less likely to interact with the negatively charged bacterial envelope (Fig. S2†).^56^ However, after prolonged exposure and a clear adaptation phenotype, it is important to evaluate if the stress of NMC has caused alterations in the membrane and cell wall structures.^57^ Untreated *S. oneidensis* MR-1 had a smooth bacterial membrane as reported previously.^12^,^14^ Cultures adapted to 25 mg L^–1^ NMC and 25 mg L^–1^ NMC metal ions show filamentation as seen in SEM images, but no significant morphological differences in the cell envelope.

### Initial mechanistic investigation of resistance: secreted biomolecules

**Fig. 8:**
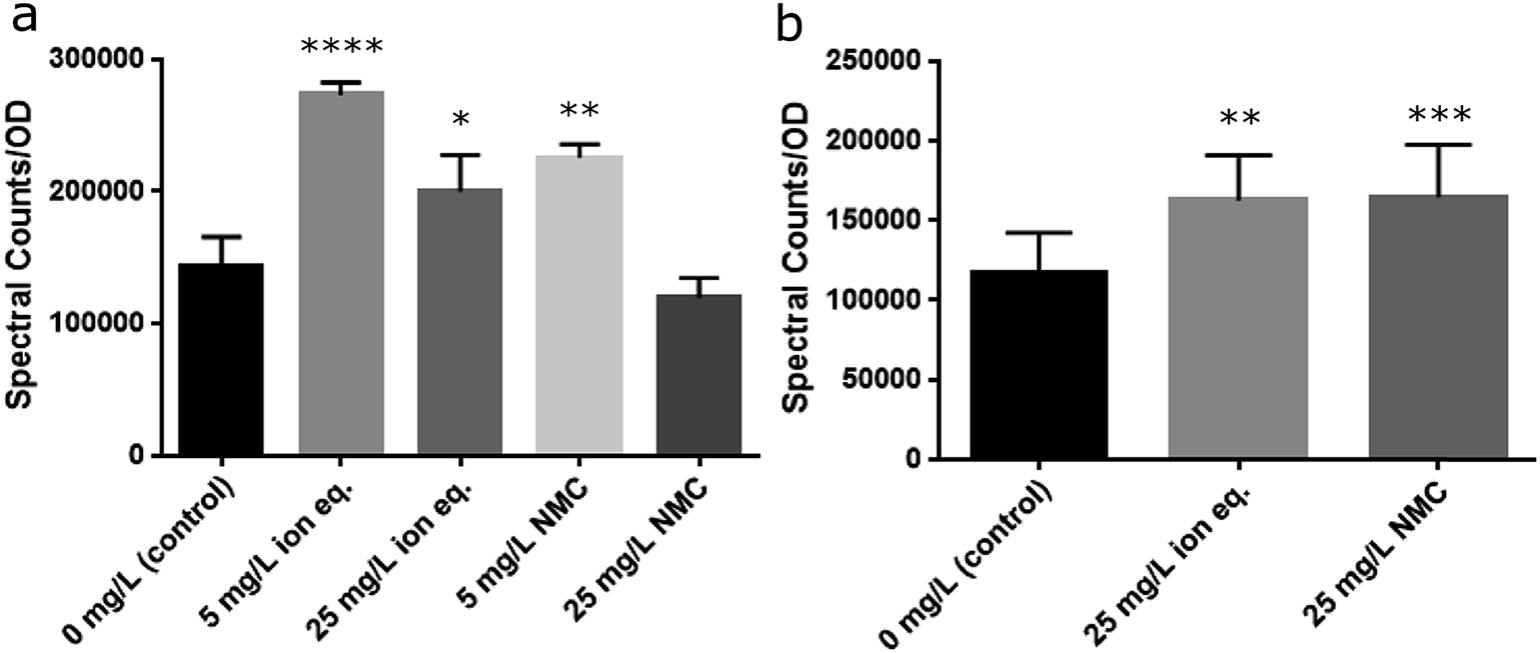
Riboflavin secretion as measured with LC-MS. (a) Passage A demonstrates significant differences between the treated groups and the control. (b) Over multiple subsequent passages (average of B–E), the control differs from the ion- and NMC-treated samples, but these samples do not differ from one another. Statistical analysis performed with non-parametric one-way ANOVA with Tukey analysis as necessary (*α* = 0.05; ****p ≤ 0.0001; ***p ≤ 0.001; **p ≤ 0.01, *p ≤ 0.05).

*S. oneidensis* MR-1 is well known for its ability to reduce a variety of extracellular substances and metals *via* secretion of riboflavin, an electron shuttle that is transported through the Mtr pathway, which is a collection of membrane-embedded cytochrome proteins.^58^,^59^ The Mtr pathway of *S. oneidensis* MR-1 has been linked to the reduction of metals and metal oxides, including manganese and cobalt under anaerobic conditions.^60^,^61^ Furthermore, riboflavin has been shown to increase in the supernatant of a culture over time, as well as after exposure to TiO_2_ nanoparticles.^58^,^62^ The modification of metal oxidation states has been identified as a mechanism of bacterial resistance to toxic metals.^63^ Due to the change in behavior of *S. oneidensis* MR-1 during repeated exposures to metals, it was considered likely that the activity of this pathway and the secreted concentration of riboflavin may increase. As such, throughout Passages A–E the supernatants were collected for riboflavin analysis by liquid chromatography-mass spectrometry (LC-MS). We observed an increase in relative riboflavin concentrations upon treatment with NMC or its ions that persisted across multiple passages, correlating with the stability of the resistance phenotype (Fig. 8 and S14†). Populations exposed to 25 mg L^–1^ of NMC during the first passage show a slight decrease in relative riboflavin concentrations when normalized to optical density. Yet, bacteria exposed to only 5 mg L^–1^ of NMC or the ion equivalents secreted more riboflavin than the control. This contrast could be due to the fitness of the populations during the initial exposure (Passage A) and indicate that more resources are allocated to other survival mechanism instead of riboflavin secretion after exposure to 25 mg L^–1^ NMC. During subsequent passages (B–E), the relative concentrations of riboflavin are significantly different from the control, but do not increase when the population is exposed to triple the quantity of NMC (Fig. S14†). Although riboflavin is associated with the ability of *S. oneidensis* MR-1 to reduce metals extracellularly under anaerobic growth, the increased concentration may also indicate some metal related-utility under aerobic respiration.

Finally, we have also confirmed that there is no secreted factor, such as a protein or small molecule, which is responsible for the ability of the organism to survive exposure. Bacteria from the control population were cultured using spent media from NMC-adapted cultures. These cultures rebounded after exposure to 5 mg L^–1^ as seen previously, but did not grow in cultures containing 10 mg L^–1^ (Fig. S15†). These data provide further evidence that the adaptation is heritable, and not triggered by media factors or secreted biomolecules.

## Summary & conclusions

Nanoparticles have been considered a potential alternative to existing antibiotic treatments. Because they typically trigger multiple mechanisms of response, it has been postulated that bacteria may not develop resistance to nanoparticles.^18^,^64^–^66^ Yet, bacteria and other microorganisms have always existed in environments that naturally contain metals, so it is not surprising that they can also adapt to the presence of metal nanoparticles.^63^ Here, we provide the first example of stable bacterial resistance to a metal nanoparticle, outside of antimicrobial silver materials, which were only recently demonstrated to spur resistance.^38^ Other studies investigating nanoparticle adaptations have shown clear bacterial response but have not demonstrated a stable nanoparticle resistance phenotype.^41^,^48^,^66^–^69^ Due to the ability of *S. oneidensis* MR-1 to continue replication under increasing concentrations of NMC, the heritability and stability of the adaptation after the pressure has been removed, and the presence of a nanoparticle-specific impact, it is rational to consider this resistance and not simply tolerance.^37^,^38^,^49^,^65^

Microbial resistance is important to consider when addressing the regulation of soluble nanomaterials. There is a “particle-chemical duality challenge” that cannot be addressed when only assessing particle dissolution, especially when considering potential toxicity.^70^ Even for nanoparticles like NMC, where metal dissolution is responsible for much of the particle toxicity, there are toxic properties unique to the particle, which have also been reported for AgNPs.^71^ Other research has shown differential toxicity between a nanomaterial and its ions, but the ions were more toxic than the nanoparticle.^38^,^72^,^73^ In this work, we saw that ions were similarly toxic to the nanomaterial, but with further culturing and adaptation development, it was possible to distinguish between the toxicity of NMC and its equivalent ions by characterizing the adapted populations. There are several mechanisms of metal resistance that a bacteria may employ to mitigate NMC toxicity, but with multiple metals and an undetermined “nanoparticle-specific factor,” elucidation of these mechanism will likely be complex.^63^ Future studies will work to determine the mechanisms of resistance and more thoroughly map the variations between ion and nanoparticle adaptation, as well as determine if nanoparticle resistance is possible in other organisms.

In sum, the presented studies make clear that it is critical to perform chronic exposure experiments when assessing the toxicity of nanomaterials as this is more similar to their environmental and medical mode of action.^64^ Such experiments enable the elucidation of permanent adaptation mechanisms, as seen here, and indicate that while many previously postulated that nanoparticles were unlikely to promote bacterial resistance due to their multiple mechanisms of action, these small organisms are readily able to adapt to such assaults. Given the essential roles that bacteria play in our ecosystems, it is clear that careful assessment of chronic exposure to engineered materials is required to avoid drastic modifications and unnecessary resistance in regions of contamination.^36^,^74^–^76^

## Materials and methods

The experimental flow chart across passages can be found in the ESI (Fig. S3).†

### NMC synthesis and characterization

Lithium nickel manganese cobalt oxide nanosheets with stoichiometric Ni : Mn : Co were synthesized as previously published.^12^ First, a nickel manganese cobalt hydroxide precursor with stoichiometric Ni : Mn was synthesized through dropwise addition of aqueous transition metal salt containing 0.2 M nickel(ii) acetate, 0.2 M manganese(ii) acetate, and 0.2 cobalt(ii) acetate into 0.1 M aqueous LiOH with stirring. The precursor was purified and isolated with multiple cycles of centrifugation with water (2×) and methanol (3×) before drying under a flow of nitrogen. The mixed metal hydroxide (0.250 g) was added to 10 g mixture of molten lithium salt flux (6 : 4 molar ratio of LiNO_3_ : LiOH) at 205 °C for 30 min with stirring. The reaction is quenched with water to yield NMC nanosheets, which were purified with cycles of centrifugation (2× water, 3× methanol) and dried under a continuous flow of nitrogen. As described in previous studies, TEM images show sheet-like morphologies that average ∼ 80 nm across and the colloidal stability and aggregation of the particles were assessed by *ζ*-potential and DLS (method fully detailed in the ESI†).^12^,^14^

To characterize metal dissolution into the bacteria media, a suspension of NMC was added to media to yield a final concentration of 5 mg L^–1^ and 25 mg L^–1^ NMC. Samples were agitated by an incubator shaker for 96 h at 30 °C. Samples were collected in triplicate and centrifuged at 4696*g* for 20 min to remove a majority of NMC in solution. The supernatant was again centrifuged to collect any remaining nanoparticles at 288 000*g* for 2 h using a Beckman Coulter Optima Ultracentrifuge with a SW-41 Ti Rotor. Resulting supernatants were measured by ICP-OES in triplicate to determine the concentration of dissolve metal species.

### Bacterial cultivation

*Shewanella oneidensis* MR-1 (ATCC BAA1096) were grown on lysogeny broth (LB) agar plates at 30 °C for 16 h and transferred in a minimal medium containing 11.6 mM NaCl, 4.0 mM KCl, 1.4 mM MgCl_2_, 2.8 mM Na_2_SO_4_, 2.8 mM NH_4_Cl, 88.1 μM Na_2_HPO_4_, 50.5 μM CaCl_2_, 10 mM HEPES, and 100 mM fresh sodium lactate. Liquid cultures were grown at 30 °C, shaking at 250 RPM, for 24 h into exponential phase. The cultures were diluted to an optical density (OD) of ∼0.2 at 600 nm (GENESYS 20 spectrophotometer, ThermoFisher Scientific).

### Bacterial exposure cultivation and analysis

Bacteria suspensions were diluted 1 : 10 v/v into fresh media. NMC nanoparticle (2 mg mL^–1^) were dispersed in minimal media with sonication for 10 min and added to the cultures to attain the desired NMC concentration either at the time of the culture dilution (time 0 h) or 10 h after dilution (time 10 h). Likewise, stock solutions of LiOH, NiCl_2_, MnSO_4_, and CoCl_2_ in minimal media were all added to the cultures at time 0 to achieve Li^+^, Ni^2+^, Mn^2+^, and Co^2+^ concentrations in the media according to the expected metal ion dissolution of NMC over 96 h as determined by ICP-OES (*vide supra*). All conditions were performed in triplicate. Bacterial growth was monitored by turbidity through optical absorbance at 600 nm every few h for 72–96 h (referred to as Passage A). A blank of minimal media with the same concentration of NMC was used.

For each subsequent exposure, the bacterial suspensions were diluted to an OD_600_ of 0.1 using minimal media without lactate and diluted 1 : 10 (v/v) into fresh media and lactate supplemented with NMC nanoparticle or metal ions at time 0 (Passages B–E). Carry-over of NMC or metal ions into subsequent cultures was considered negligible since the suspensions were diluted 20–30 times when in subsequent culture. After 96 h, an aliquot from each culture from Passages A–E was collected for riboflavin analysis (*vide infra*).

### Respirometry experiments

Passage E was used for respirometry experiments. Bacterial respiration, as a measure of bacteria fitness during exposure to NMC or metal ions, was determined by quantifying O_2_ (g) consumption using a 24-vessel respirometer system (Respirometry Systems and Applications, Inc., Springdale, AR). Bacteria from Passage D were diluted to create a 100 mL suspension with a final OD_600_ of 0.01 for Passage E growth. Cultures were placed in a 125 mL glass vessel and supplemented with NMC and metal ions. The vessels were placed in a 30 °C water bath for 72 h with constant stirring (500 rpm) and under constant O_2_ pressure. The CO_2_ produced by the respiring bacteria was scrubbed with a KOH insert in the headspace of the vessel. As CO_2_ was removed from the gas phase, O_2_ was delivered to the vessel every 5 min to maintain a constant pressure. The total mass of O_2_ delivered to the system and the time it took to reach the highest rate of respiration (as determined by the first derivative of the respiration curve) were used as a measure of bacterial fitness and metabolic activity.

### Riboflavin analysis

Aliquots of cultures from Passages A–E were collected at the end of each growth curve and centrifuged to remove bacteria (3220*g*, 10 min). Supernatants (2 μL injected) were analyzed on an UHD Accurate-Mass Q-TOF LC/MS instrument (Agilent, 6540), separated on a reverse phase C_18_ column (Agilent, Eclipse Plus, 2.1 × 50 mm, 1.8 μm), and detected by electrospray ionization (positive ion mode). Sample were separation was initiated with an isocratic elution of 100% A at 0.40 mL min^–1^ for 2 min followed by a linear gradient of 0–100% B over 7 min, then an isocratic elution at 100% B for 1 min (A: 95 : 5H_2_O : ACN, 0.1% formic acid; B: 95 : 5 ACN : H_2_O, 0.1% formic acid).

### Bacteria morphology analysis with electron microscopy

All SEM samples from Passage D were prepared by pelleting from growth media and washing with 1× DPBS (10 mL, 750*g*), followed by a wash with 2 mM HEPES buffer (10 mL, 750*g*). Samples were then fixated with 5% glutaraldehyde (500 μL), with an incubation of 30 min, followed by two washes of 0.1 M phosphate buffer (500 μL, 1200*g*). Cells underwent dehydration by ethanol in successive steps of increase ethanol concentrations (35%, 50%, 75%, 95%, 100%; 500 μL, 1200*g*). Final dehydration occurred by two washes with HDMS (500 μL, 800*g*). Cells from the second HDMS wash were allowed to desiccate directly onto glass cover slides. Sample slides were sputter coated with a thin layer of gold to increase conductivity just before being placed into the SEM for imaging. Samples were imaged on a JEOL JSM-IT100 SEM at a working distance of 10 mm with a probe current of 0.045 μA and electron gun voltage of 5 kV.

### Analysis of SEM images

Dimensions of Passage D bacteria analyzed were blinded in SEM preparation and analysis in ImageJ. Using ImageJ, each cell's width and length was measured, after the image was calibrated using the scale bar. The cell length was measured by using the segmented line tool down the center of the bacteria. The width was measured with the straight line tool on a portion of the bacteria where the cell membrane was intact and not curving. A cell was only measured if its start and end was clearly defined. The growth conditions of each sample were double blinded before the bacteria were measured.

## Conflicts of interest

There are no conflicts to declare.

## Supplementary Material

Supplementary information
