# Supplementary material for: Chronic exposure to complex metal oxide nanoparticles elicits rapid resistance in Shewanella oneidensis MR-1
Source: Chem Sci. 2019 Aug 30;10(42):9768–81. doi: 10.1039/c9sc01942a (PMC6993611; doi:10.1039/c9sc01942a)
Supplement: Supplementary file 1 [file SC-010-C9SC01942A-s001.pdf]

Electronic Supplementary Material (ESI) for Chemical Science.
This journal is © The Royal Society of Chemistry 2019

# *Supporting Information for*

## Chronic exposure to complex metal oxide nanoparticles elicits rapid resistance in *Shewanella oneidensis* MR-1

Stephanie L. Mitchell,[1] Natalie V. Hudson-Smith,[1] Meghan S. Cahill,[1] Benjamin N. Reynolds,[2] Seth D. Frand,[3] Curtis M. Green,[4] Chenyu Wang,[4] Mimi N. Hang,[4] Rodrigo Tapia Hernandez,[3] Robert J. Hamers,[4] Z. Vivian Feng,[3] Christy L. Haynes,[1] Erin E. Carlson[1,2,5]*

[1]Department of Chemistry, University of Minnesota, 207 Pleasant St. SE, Minneapolis, MN 55455
[2]Department of Biochemistry, Molecular Biology, and Biophysics, University of Minnesota, 321 Church Street SE, Minneapolis, Minnesota 55454
[3] Chemistry Department, Augsburg University, 2211 Riverside Ave, Minneapolis, MN 55454
[4] Department of Chemistry, University of Wisconsin-Madison, 1101 University Avenue, Madison, WI 53706
[5]Department of Medicinal Chemistry, University of Minnesota, 208 Harvard Street SE, Minneapolis, Minnesota 55454

**Materials and Methods**

NMC Characterization with TEM, ζ-potential, and DLS

MIC and $MDK_{99}$ Assays

Media Memory

Bacterial membrane analysis with electron microscopy

Analysis of TEM images

**List of Figures**

**Figure S1.** Size analysis of NMC.

**Figure S2.** DLS and ζ-potential characterization of NMC.

**Figure S3.** Schematic summary of experiments, both simplified and detailed.

**Figure S4.** Dissolved ion concentrations from NMC dissolution.

**Figure S5.** Statistical analysis of growth curves.

**Figure S6.** Optical density growth curves from Passage E.

**Figure S7.** First derivative of respirometry plots and respirometry plots without error bars.

**Figure S8.** Comparison of exposure conditions across passages.

**Figure S9.** Optical density growth curves of increase concentrations of NMC.

**Figure S10.** MIC measurements of control and resistant bacteria.

**Figure S11.** $MDK_{99}$ time course.

**Figure S12.** Histogram of bacterial lengths and summary of bacterial lengths and widths from SEM.

**Figure S13.** TEM of passaged bacteria.

**Figure S14.** Riboflavin quantity with increasing concentrations of NMC and fragmentation data.

**Figure S15.** Optical density growth curves to show impact of spent media.

**NMC Characterization with TEM, ζ-potential, and DLS**

The morphology and size of NMC nanoparticles was analyzed using a transmission electron microscope (TEM). NMC nanoparticles were suspended in methanol (Sigma) at a concentration of 100 mg/L via ultrasonication. The solution was then drop-cast onto a 200 mesh Cu TEM grid with a pure carbon film support (Ted Pella). The particles were imaged using a FEI Tecnai T12, operated at an accelerating voltage of 120 kV. Images were analyzed using ImageJ software.

The aggregation state and stability of NMC nanoparticles in minimal media with lactate were analyzed by dynamic light scattering (DLS) and laser doppler microelectrophoresis measurements taken with a Malvern Zetasizer Nano-ZS. A 2,000 mg/L stock solution of NMC was prepared in 25 mL of minimal media with lactate and bath sonicated for 10 minutes. 15 aliquots of the stock solution were removed and diluted to 25 mg/L, and the samples were placed on a Thermolyne Rotomix 51300 Orbital Shaker operating at 200 rpm. Samples were removed from the shaker immediately prior to analysis of nanoparticle diffusion coefficient via DLS and ζ-potential by laser doppler microelectrophoresis. Aliquots from the samples were removed from the top of the samples and added into the cuvette for analysis. Analysis timepoints were 0 h, 24 h, 48 h, 72 h, and 96 h. The results reported are the averages and standard deviations of 3 sample replicates, with five DLS and five ζ-potential measurements made per sample.

**MIC and $MDK_{99}$ Assays**

In Passage D, sensitive and resistant *S. oneidensis* (0.01 OD, 5 mL cultures) were exposed to ten increasing concentrations of lithium (6,000–12,000 μM for control; 6,000-24,000 μM for NMC-resistant), nickel (30–80 μM for control; 40–220 μM for NMC-resistant), manganese (4,000–9,400 μM for control; 4,000–22,000 μM for NMC-resistant), and cobalt (20–65 μM for

control; 20–200 μM for NMC-resistant) from stock solutions of LiOH, $NiCl_2$, $MnSO_4$, and $CoCl_2$. After 72 h of growth, cultures were analyzed for the lowest concentration that prevented bacterial growth, which was determined to be the MIC value.

Additionally, during Passage D, an $MDK_{99}$ assay was performed. Sensitive *S. oneidensis* were exposed to 0, 5, 10, and 25 mg/L NMC and NMC-resistant *S. oneidensis* was exposed to 25 mg/L NMC at an OD of 0.01 (~1 x $10^7$ CFU per mL) as done in previous passages. Every 2-5 h, 100 μL of the four conditions was diluted 0-100,000x and each dilution was dropped in six 10 μL aliquots onto an LB agar plate, prepared by drying for 1 h at 30 °C and irradiating with UV light for 15 minutes. After the drops were fully absorbed into the agar, the plates were incubated at 30 °C for 24 h, and the bacteria were counted to determine the CFU per mL.

**Media memory**

During Passage D, the media was tested to see if there were any signaling molecules in the media that might be responsible for triggering the adaptation phenotype through subculturing. After diluting the control bacteria and NMC-adapted bacteria to the same OD (~0.1), an aliquot of the NMC-adapted bacteria suspension was centrifuged and filtered to remove any bacteria. Supernatant from NMC-adapted cultures (0.5 mL) and control bacteria suspension (0.5 mL) was placed into fresh minimal media (4 mL) with the final concentration of lactate being ~100 mM (assuming the cultured media is lacking lactate). These cultures were placed into increasing concentrations of NMC and were compared to control cultures without NMC-adapted media exposed to identical concentrations of NMC.

**Bacterial membrane analysis with electron microscopy**

A sample from Passage C was collected at 48 h (late stationary phase). The bacteria were collected by centrifugation (800*g*, 5 min) and washed with HEPES buffer then in 0.1 M sodium

cacodylate buffer three times before being fixed in 2.5% glutaraldehyde in 0.1 M sodium cacodylate for 50 min at RT. Pellets were washed with 0.1 M sodium cacodylate buffer three times and dehydrated with washes of increasing concentrations of ethanol (30%, 50%, 70%, 80%, 95%, and 100%). After three washes with propylene oxide, 2 h incubation in 2:1 propylene oxide:resin, overnight incubation in 1:1 propylene oxide:resin, 4 h incubation with fresh 1:1 propylene oxide:resin, the pellet was incubated in resin overnight. Resin was replaced with fresh resin and cured at 40 °C for 24 h then at 60 °C for 48 h. The resin was cut into slices (~70 nm thick) using a Leica EM UC6 ultramicrotome, stained with uranyl acetate and lead citrate for contrast, and placed on copper grids (200 mesh) with carbon and formvar supports. Bacteria were imaged on a FEI Tecnai T12 transmission electron microscope at 120 kV.

**Analysis of TEM Images**

Dimensions of Passage C bacteria analyzed with TEM were measured in ImageJ. Criterion for measurement were as follows; if the membrane was clear from all directions, the bacteria were measured for length and width. Cross-sections that were perpendicular to the slicing direction (having an aspect ratio approximately equal to 1) were kept in a separate data pool from cross-sections that were parallel to the slicing direction.

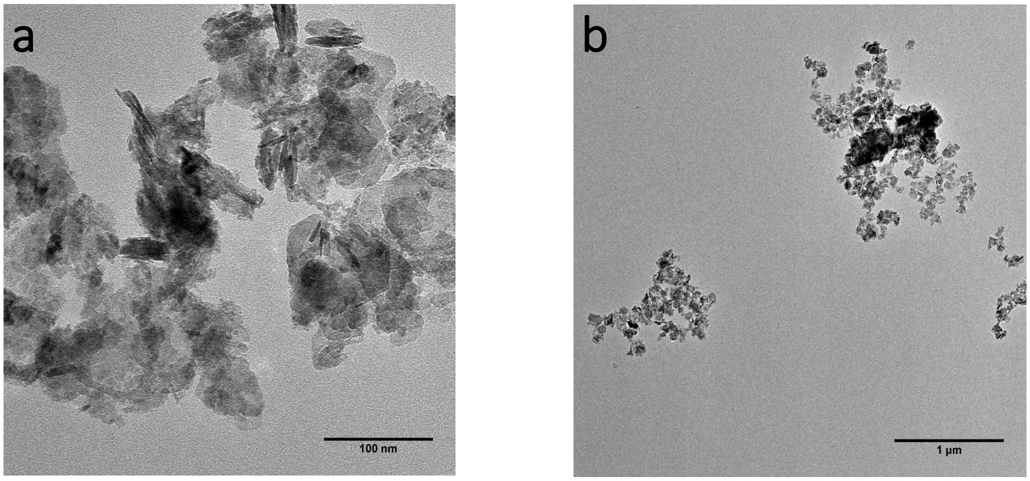


**Figure S1.** Size analysis of NMC nanoparticles. (a & b) Transmission electron microscope image of NMC.

| | 0 h | 24 h | 48 h | 72 h | 96 h |
|---|---|---|---|---|---|
| **Hydrodynamic diameter (nm)** | 2682 ± 740.7 | 1422 ± 677.5 | 1236 ± 1245 | 99.22 ± 263.8 | 1.667 ± 4.199 |
| **ζ Potential (mV)** | -10.3 ± 0.547 | -9.14 ± 1.31 | -8.29 ± 1.39 | -4.91 ± 1.96 | -2.43 ± 1.18 |

**Figure S2.** DLS and ζ-potential characterization of NMC in minimal media over time.

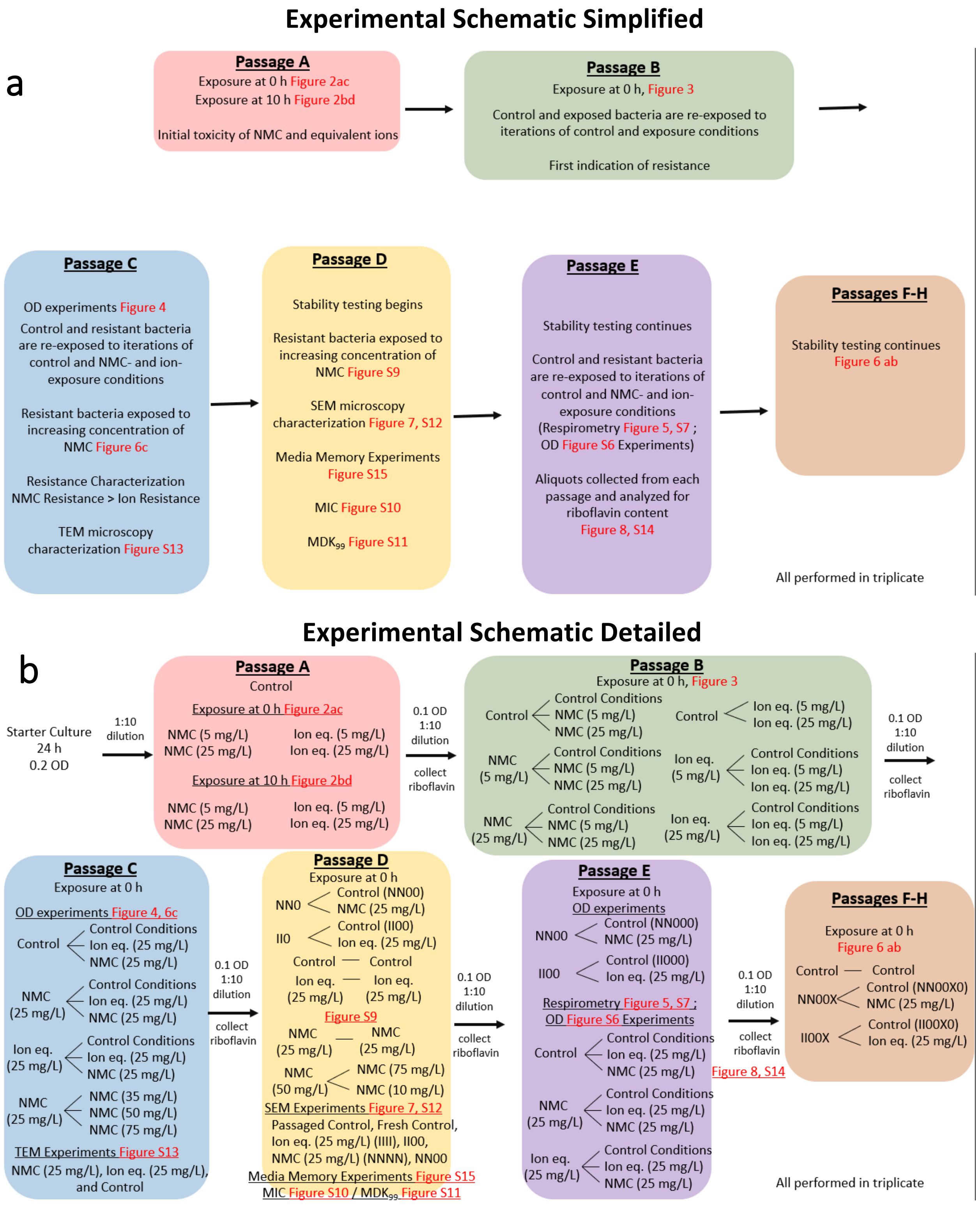


**Figure S3.** Schematic summary of the experiments conducted in this work in each passage, both (a) simplified and (b) detailed.

| NMC mass concentration (mg/L) | Dissolved metal concentration after 96 hrs (μM) | | | |
|---|---|---|---|---|
| | Ni | Co | Mn | Li |
| 5 | 8.1 ± 0.5 | 4.1 ± 0.3 | 3.4 ± 0.5 | 28.3 ± 4.9 |
| 25 | 25.8 ± 1.5 | 10.0 ± 0.7 | 6.0 ± 0.7 | 149.5 ± 6.4 |

**Figure S4.** Measured concentrations of ions produced in minimal media with lactate through dissolution of NMC. The mean and standard deviation are of four replicate samples.

a

| Passage A | | | |
|---|---|---|---|
| | μ | Max. OD | λ |
| **Control** | 0.214 | 0.229 ± 0.012 | 4 |
| **NMC (5 mg/L)** | 0.219 | 0.203 ± 0.008 | 4 |
| **NMC (25 mg/L)** | 0.190 | 0.150 ± 0.002 | 4 |
| **Ion (5 mg/L)** | 0.197 | 0.188 ± 0.002 | 4 |
| **Ion (25 mg/L)** | 0.190 | 0.150 ± 0.003 | 4 |

b

| Passage B | | | |
|---|---|---|---|
| | μ | Max. OD | λ |
| **Control > Control** | 0.315 | 0.249 ± 0.007 | 17 |
| **Control > NMC (5 mg/L)** | 0.000 | 0.000 ± 0.000 | x |
| **Control > NMC (25 mg/L)** | 0.000 | 0.000 ± 0.000 | x |
| **NMC (5 mg/L) > Control** | 0.188 | 0.256 ± 0.013 | 19.75 |
| **NMC (5 mg/L) > NMC (5 mg/L)** | 0.072 | 0.240 ± 0.016 | 60.5 |
| **NMC (5 mg/L) > NMC (25 mg/L)** | 0.000 | 0.000 ± 0.000 | x |
| **NMC (25 mg/L) > Control** | 0.120 | 0.315 ± 0.008 | 38.25 |
| **NMC (25 mg/L) > NMC (5 mg/L)** | 0.151 | 0.278 ± 0.013 | 48.75 |
| **NMC (25 mg/L) > NMC (25 mg/L)** | 0.109 | 0.185 ± 0.008 | 60.5 |
| **Control > Ion (5 mg/L eq.)** | 0.000 | 0.000 ± 0.000 | x |
| **Control > Ion (25 mg/L eq.)** | 0.000 | 0.000 ± 0.000 | x |
| **Ion (5 mg/L eq.) > Control** | 0.156 | 0.256 ± 0.005 | 22.75 |
| **Ion (5 mg/L eq.) > Ion (5 mg/L eq.)** | 0.093 | 0.247 ± 0.006 | 60.5 |
| **Ion (5 mg/L eq.) > Ion (25 mg/L eq.)** | 0.130 | 0.163 ± 0.035 | 77 |
| **Ion (25 mg/L eq.) > Control** | 0.147 | 0.294 ± 0.004 | 29.75 |
| **Ion (25 mg/L eq.) > Ion (5 mg/L eq.)** | 0.139 | 0.259 ± 0.004 | 48.75 |
| **Ion (25 mg/L eq.) > Ion (25 mg/L eq.)** | 0.100 | 0.248 ± 0.004 | 57 |

c

| Passage C | | | |
|---|---|---|---|
| | μ | Max. OD | λ |
| **Control > Control** | 0.141 | 0.252 ± 0.007 | 9 |
| **Control > NMC (25 mg/L)** | 0.000 | 0.000 ± 0.000 | x |
| **Control > Ion (25 mg/L eq.)** | 0.000 | 0.000 ± 0.000 | x |
| **NMC (25 mg/L) > Control** | 0.261 | 0.331 ± 0.006 | 36.25 |
| **NMC (25 mg/L) > Ion (25 mg/L eq.)** | 0.159 | 0.262 ± 0.028 | 42.5 |
| **NMC (25 mg/L) > NMC (25 mg/L)** | 0.124 | 0.230 ± 0.018 | 45.5 |
| **Ion (25 mg/L eq.) > Control** | 0.234 | 0.318 ± 0.004 | 26 |
| **Ion (25 mg/L eq.) > Ion (25 mg/L eq.)** | 0.177 | 0.263 ± 0.027 | 33.75 |
| **Ion (25 mg/L eq.) > NMC (25 mg/L)** | 0.129 | 0.266 ± 0.008 | 36.25 |

d

| Passage H | | | |
|---|---|---|---|
| | μ | Max. OD | λ |
| **IIXI** | 0.093 | 0.391 ± .013 | 10 |
| **IIX0** | 0.122 | 0.400 ± 0.005 | 10 |
| **NNXN** | 0.085 | 0.349 ± 0.025 | 10 |
| **NNX0** | 0.095 | 0.353 ± 0.012 | 10 |
| **Control** | 0.067 | 0.336 ± 0.016 | 10 |
| **NMC** | 0.060 | 0.351 ± 0.021 | 10 |
| **Ion** | 0.076 | 0.393 ± 0.020 | 10 |

**Figure S5.** Comparison of the specific growth rate (μ) of the exponential phase of the growth curves, the highest OD achieved in stationary phase, representing the total number of bacteria, as well as the lag time in h (λ) for Passages A, B, C, and H (parts a, b, c, and d, respectively). (a) Bacteria exposed at time 10 h. (d) 'X' represents 5 passages without exposure to NMC or ion equivalent, 'Ion' indicates bacteria cultured in 8 passages exposed to 25 mg/L ion equivalent, 'NMC' indicates bacteria cultured in 8 passages exposed to 25 mg/L NMC, and 'Control' indicates bacteria cultured without NMC or ions for 8 passages.

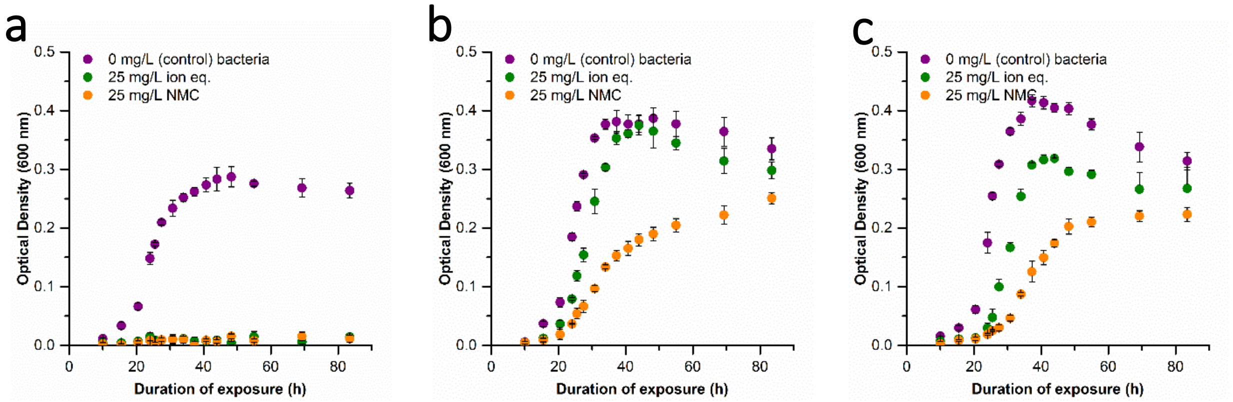


**Figure S6.** Effect of repetitive NMC and ion exposure on growth inhibition of *S. oneidensis* (Passage E). (a) Bacteria cultured for four passages in pristine media exposed to no NMC (purple), 25 mg/L NMC ion eq. (green), and 25 mg/L NMC (orange). (b) Bacteria cultured for four passages with 25 mg/L NMC exposed to no NMC (purple), 25 mg/L NMC ion eq. (green), and 25 mg/L NMC (orange). (c) Bacteria cultured for four passages with 25 mg/L NMC ion eq. exposed to no NMC (purple), 25 mg/L NMC ion eq. (green), and 25 mg/L NMC (orange). Error bars represent the standard deviation of three replicates.

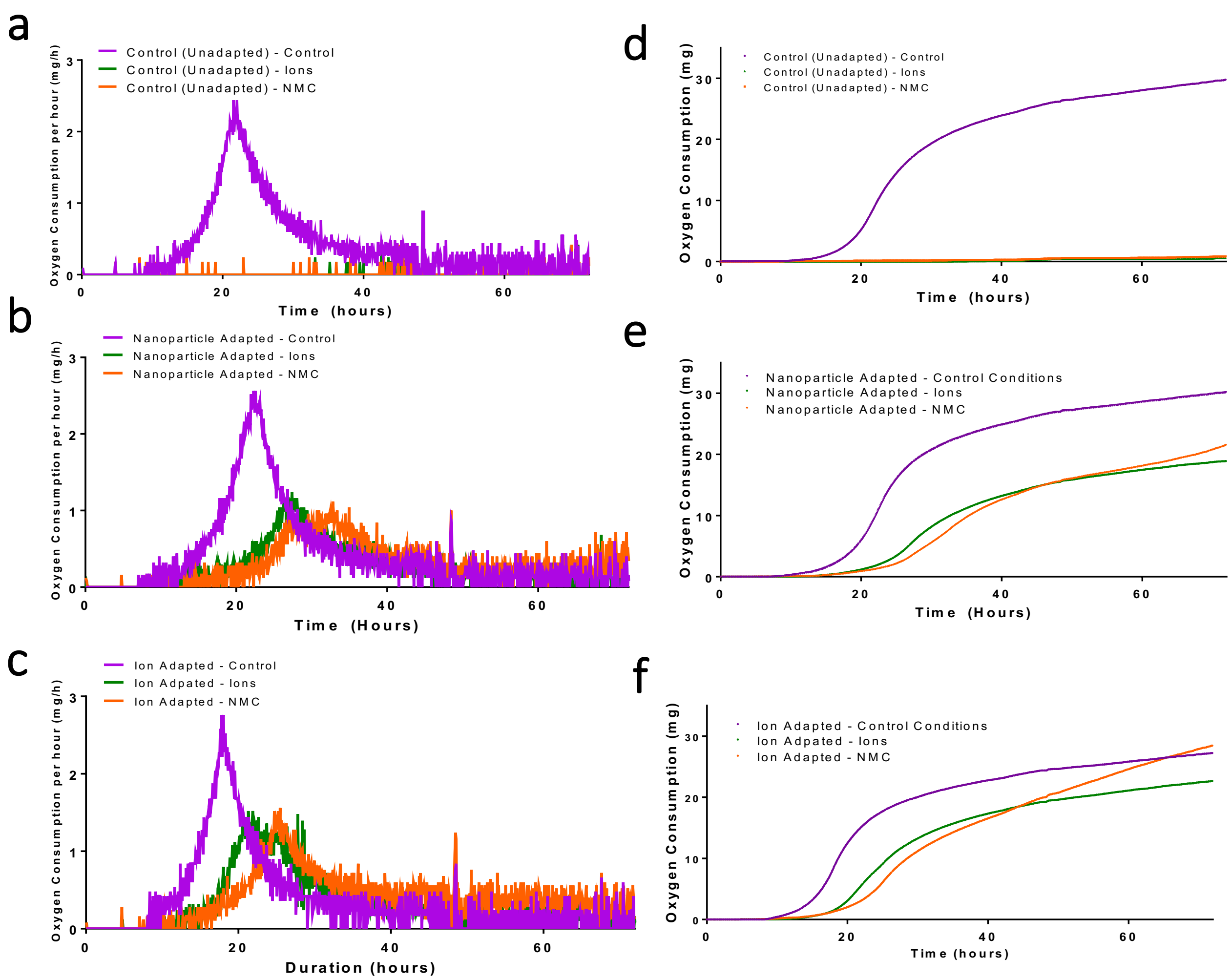


**Figure S7.** First derivative of respirometry growth curves represented in Figure 5. (a) First derivatives of *S. oneidensis* MR-1 control (unadapted) cultures exposed to control conditions (purple), 25 mg/L NMC (orange), and 25 mg/L NMC-equivalent ions (green) in Passage E. (b) First derivatives of nanoparticle-adapted cultures of *S. oneidensis* MR-1 exposed to control conditions (purple), 25 mg/L NMC (orange), and 25 mg/L NMC-equivalent ions (green) in Passage E. (c) First derivatives of ion-adapted cultures of *S. oneidensis* MR-1 exposed to control conditions (purple), 25 mg/L NMC (orange), and 25 mg/L NMC-equivalent ions (green) in Passage E. An alternative representation of data from Figure 4 without standard deviations for clearer graphics. (d) Respirometry growth curves of *S. oneidensis* MR-1 control (unadapted)

cultures exposed to control conditions (purple), 25 mg/L NMC (orange), and 25 mg/L NMC-equivalent ions (green) in Passage E. (e) Respirometry growth curves of nanoparticle-adapted cultures of *S. oneidensis* MR-1 exposed to control conditions (purple), 25 mg/L NMC (orange), and 25 mg/L NMC-equivalent ions (green) in Passage E. (f) Respirometry growth curves of ion-adapted cultures of *S. oneidensis* MR-1 exposed to control conditions (purple), 25 mg/L NMC (orange), and 25 mg/L NMC-equivalent ions (green) in Passage E.

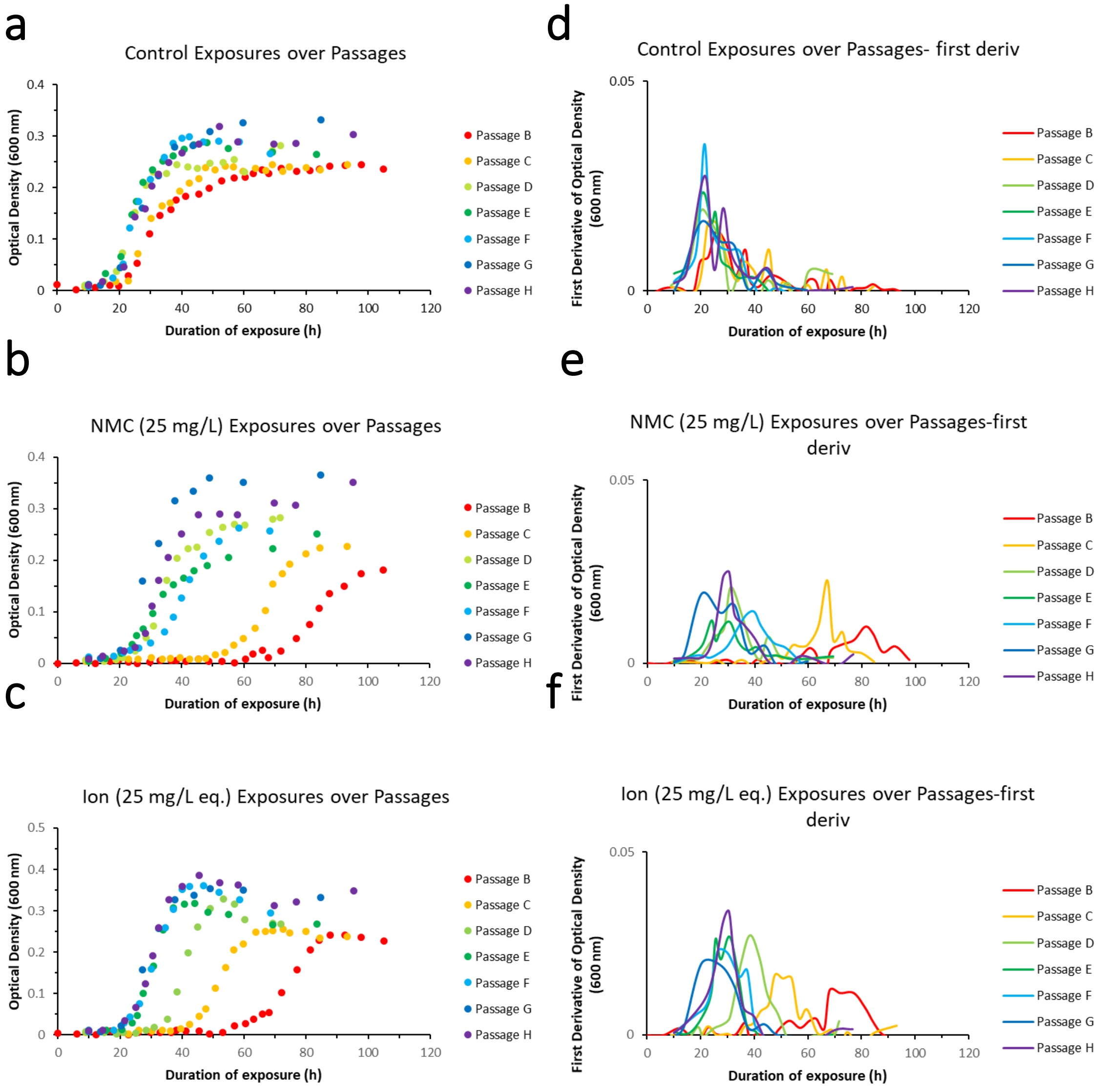


**Figure S8.** Bacteria cultured in (a) control conditions (b) NMC (25 mg/L) and (c) NMC-equivalent ions (25 mg/L) over Passages B-H. The first derivative of these curves (d) control conditions (e) NMC (25 mg/L) and (f) NMC-equivalent ions (25 mg/L) over Passages B-H shows the peak growth of *S. oneidensis* during the exponential phase.

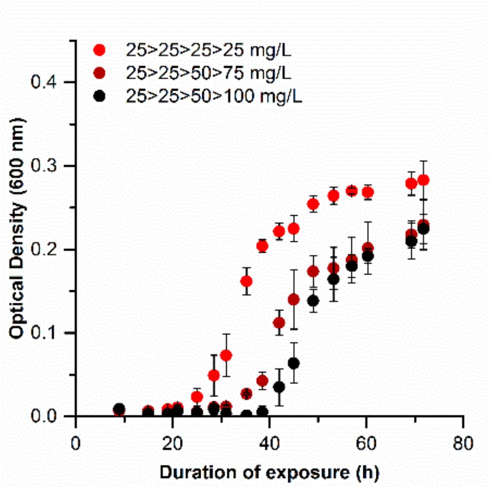


**Figure S9.** Bacteria cultured for two passages in 25 mg/L NMC exposed to increasing concentrations of NMC (Passage D). Error bars represent the standard deviation of three replicates.

| Dissolved metal concentration (µM) | Control Bacteria | Resistant Bacteria |
|---|---|---|
| Li | 9,600 ± 290 | 9,000 ± 0 |
| Ni | 50 ± 0 | 210 ± 6 |
| Mn | 7,000 ± 0 | 6,800 ± 290 |
| Co | 45 ± 0 | 160 ± 10 |

**Figure S10.** Minimum inhibitory concentrations (MICs) of lithium, nickel, manganese, and cobalt for control (unadapted) and NMC-resistant bacteria, in triplicate.

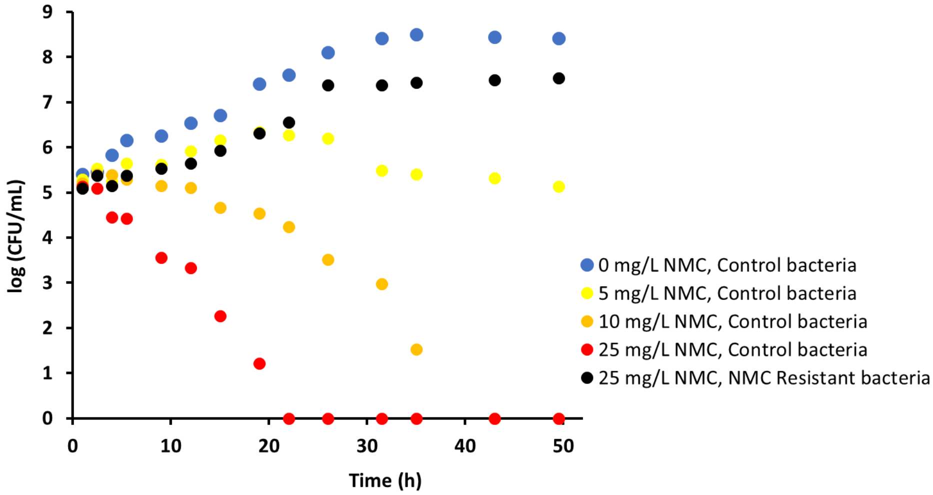


**Figure S11.** Minimum duration of killing ($MDK_{99}$) for control bacteria exposed to increasing concentrations of NMC, as well as NMC-resistant bacteria exposed to NMC (25 mg/L).

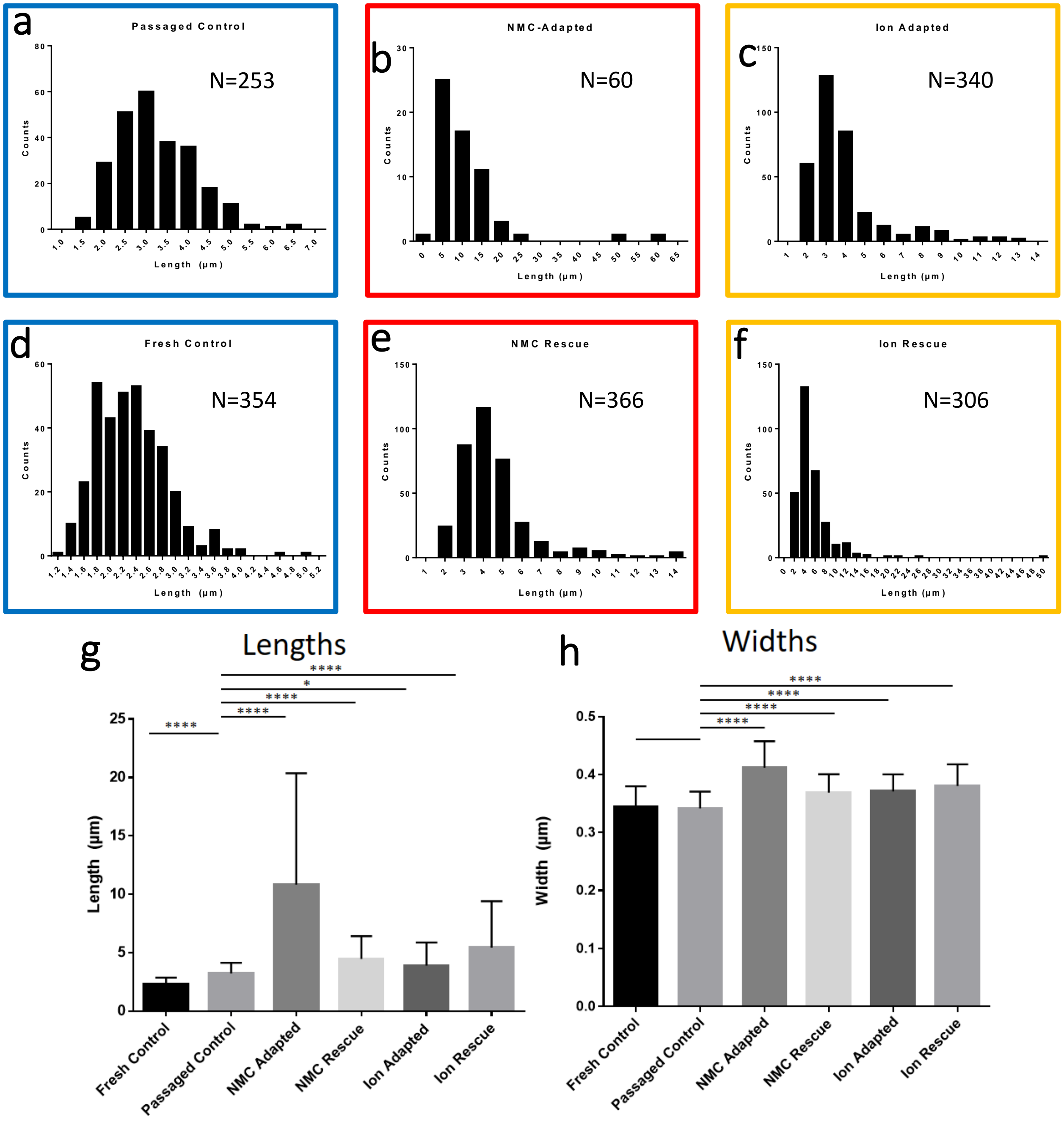


**Figure S12.** Histograms of lengths of *S. oneidensis* MR-1 from Passage D under different conditions of adaption as measured from SEM micrographs. a) no nanoparticle or ions over 4 passages, b) 25 mg/L NMC over 4 passages c) 25 mg/L NMC ion eq. over 4 passages d) no nanoparticle or ions for one passage, e) 25 mg/L NMC over 2 passages followed by 2 passages without exposure, f) 25 mg/L NMC ion eq. over 2 passages followed by 2 passages without

exposure. The averages and standard deviations of the (g) lengths and (h) widths of the bacteria from SEM micrographs. Statistical analysis performed with non-parametric one-way ANOVA with a post-hoc Tukey analysis to compare the passaged control to the other conditions. ($\alpha = 0.05$; ****$p \leq 0.0001$, ***$p \leq 0.001$; **$p \leq 0.01$, *$p \leq 0.05$).

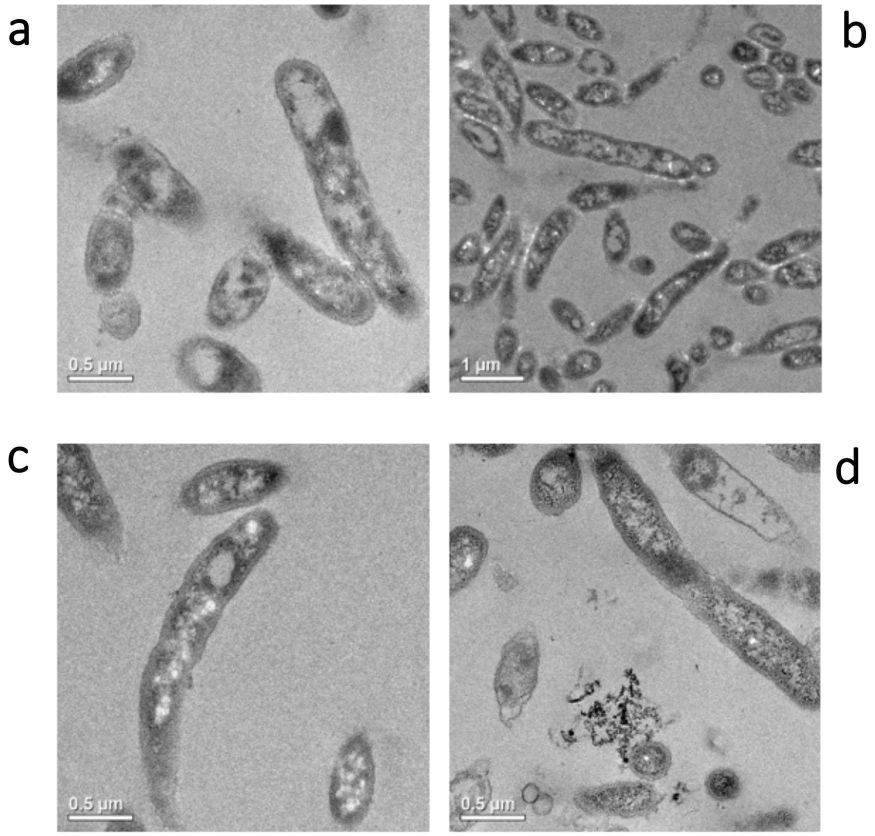


**Figure S13.** TEM micrographs of resin embedded *S. oneidensis* MR-1 under (a) control conditions from fresh culture, (b) passaged controls, (c) adapted to NMC-equivalent ions, and (d) adapted to NMC exposure from Passage D.

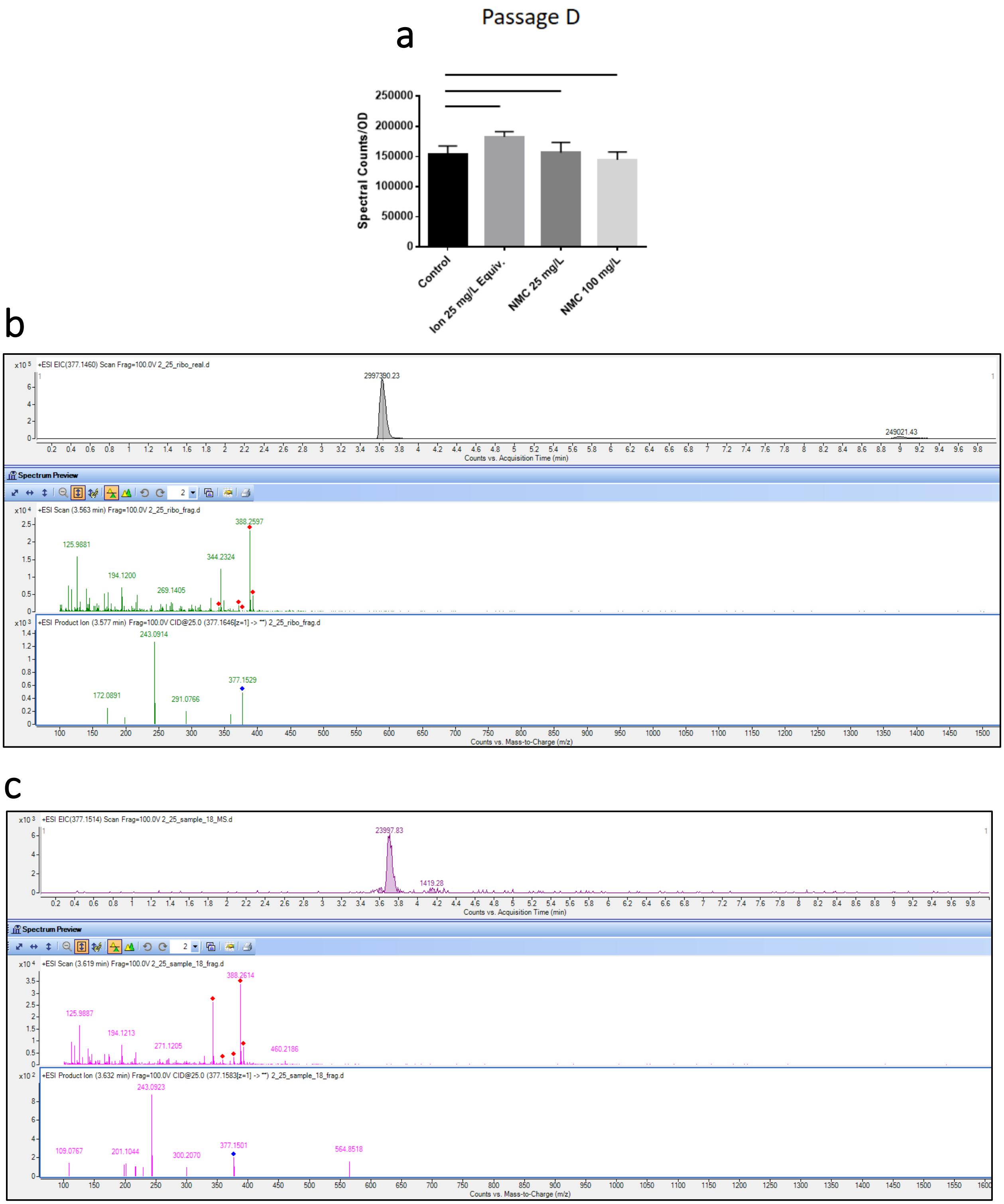


**Figure S14.** (a) Riboflavin secretion as measured with LC-MS in Passage D. Statistical analysis performed with non-parametric one-way ANOVA with a post-hoc Tukey analysis to compare the passaged control to the other conditions. (b) Fragmentation of riboflavin standard (c)

Fragmentation of sample feature also with *m/z* of 377.15 that shows characteristic fragment with *m/z* of 243.09.

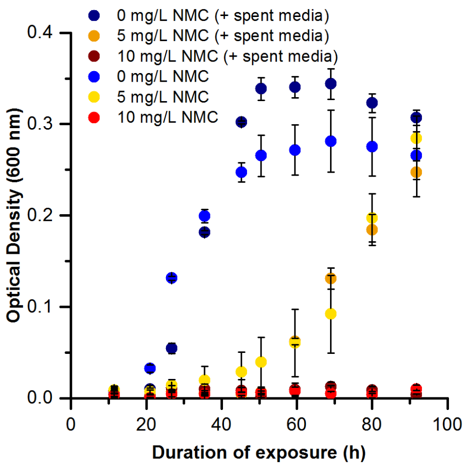


**Figure S15.** Unadapted *S. oneidensis* sub-cultured using pristine media, exposed to 0, 5, 10 mg/L NMC, compared to unadapted *S. oneidensis* subcultured using spent media from NMC-adapted bacteria, exposed to 0, 5, 10 mg/L NMC. Error bars represent the standard deviation of three replicates.
